# Effect of pH on the influenza fusion peptide properties unveiled by constant-pH molecular dynamics simulations combined with experiment

**DOI:** 10.1038/s41598-020-77040-y

**Published:** 2020-11-18

**Authors:** Diana Lousa, Antónia R. T. Pinto, Sara R. R. Campos, António M. Baptista, Ana S. Veiga, Miguel A. R. B. Castanho, Cláudio M. Soares

**Affiliations:** 1 ITQB NOVA, Instituto de Tecnologia Química e Biológica António Xavier, Universidade Nova de Lisboa, Av. da República, 2780-157 Oeiras, Portugal; 2 Instituto de Medicina Molecular, Faculdade de Medicina da Universidade de Lisboa, Av. Professor Egas Moniz, 1649-028 Lisboa, Portugal

**Keywords:** Biochemistry, Biophysics, Computational biology and bioinformatics, Molecular medicine, Chemistry

## Abstract

The influenza virus fusion process, whereby the virus fuses its envelope with the host endosome membrane to release the genetic material, takes place in the acidic late endosome environment. Acidification triggers a large conformational change in the fusion protein, hemagglutinin (HA), which enables the insertion of the N-terminal region of the HA2 subunit, known as the fusion peptide, into the membrane of the host endosome. However, the mechanism by which pH modulates the molecular properties of the fusion peptide remains unclear. To answer this question, we performed the first constant-pH molecular dynamics simulations of the influenza fusion peptide in a membrane, extending for 40 µs of aggregated time. The simulations were combined with spectroscopic data, which showed that the peptide is twofold more active in promoting lipid mixing of model membranes at pH 5 than at pH 7.4. The realistic treatment of protonation introduced by the constant-pH molecular dynamics simulations revealed that low pH stabilizes a vertical membrane-spanning conformation and leads to more frequent contacts between the fusion peptide and the lipid headgroups, which may explain the increase in activity. The study also revealed that the N-terminal region is determinant for the peptide’s effect on the membrane.

## Introduction

Influenza infections affect a very large number of individuals every year and represent a serious social and economic burden^1^. The situation becomes even more dramatic when a new pandemic arises, which can lead to very high mortality rates^1^. Currently there is no universal and effective therapy against this virus and, thus, it is crucial to obtain a detailed understanding of the infectious process and its key players. One important step of this process is the fusion between the viral and host membranes, catalyzed by the fusion protein hemagglutinin, which is one of the most promising drug targets against this virus^2^. Hemagglutinin is a homotrimer and each monomer is composed of two polypeptide chains, named HA1 and HA2, connected by a disulfide bond. HA1 is responsible for binding to the sialic acid receptors on the host membrane, whereas HA2 contains the fusion machinery^3^. After binding to the receptors on the host cell, the influenza virus is uptaken by endocytosis. Release of the genetic material of the virus into the host cell occurs at the endosome membrane level. At the late endosomes there is a pH drop to a value of around 5 that triggers a large conformational change of HA, which is crucial for the fusion process^3^.

The first 23 amino acid residues of HA2 are particularly important in the fusion process, since this region inserts into the host membrane, promoting fusion and is, therefore, known as the fusion peptide^4^. This region is very conserved across different hemagglutinin subtypes (18 out of 23 residues are strictly conserved among all influenza A strains) and several mutations have been shown to abolish or impair its function^4^. Several in-vitro studies have shown that the isolated fusion peptide promotes lipid mixing of large unilamellar vesicles, which evidences that the peptide induces hemifusion, even in the absence of the rest of hemagglutinin^5–8^. The peptide structure in detergent micelles has been analyzed by NMR studies; it has a helix-turn-helix structure in which the angle between the helices depends on the peptide length^9–12^. A 20-residue long fusion peptide (HAfp-20) adopts an open inverted-V structure at pH 5^9^, whereas a 23-residue long peptide (HAfp-23) displays a more closed helical-hairpin structure, both at pH 4 and 7.4^10^.

A crucial issue that remains unclear is the orientation adopted by the fusion peptide in the membrane bilayer, as it is determinant for peptide-induced perturbation of lipid bilayers^13^. Based on the NOE interactions between the amide protons of the HAfp-23 and dodecylphosphocholine micelle protons, Lorieau et al*.* concluded that one of the peptide sides is exposed to water and postulated that the peptide adopts an interfacial conformation^10^. However, since their results were obtained in a micelle environment, which has a very different structure from a membrane bilayer, having a single lipidic layer and a considerably higher curvature, it is not clear if the peptide adopts this type of arrangement in the membrane. Molecular dynamics (MD) simulations performed by us, indicated that the peptide can adopt two different conformations in a membrane bilayer: an interfacial orientation, parallel to the membrane surface and a membrane-spanning orientation, perpendicular to the bilayer^14^. Subsequent simulation studies by Worch et al. using temperature replica exchange molecular dynamics also found that the influenza fusion peptide can adopt these two configurations and indicate that he membrane-spanning configuration corresponds to the lowest free energy minimum for the 23-residue long fusion peptide with a charged N-terminus^15,16^.

The mechanism by which the peptide induces lipid mixing is not fully elucidated, but several recent experimental and computational studies have suggested different modes of action, including altering the membrane curvature, increasing or decreasing lipid order or inducing pore formation and stabilization^14–26^. A mechanism that has been proposed based on molecular dynamics (MD) simulations asserts that lipid tail protrusion (i.e. a lipid acyl chain that extends to and beyond the corresponding phosphate group) is a determinant step in membrane fusion^19^. The occurrence of lipid tail protrusion has been observed in several simulation studies of the influenza fusion peptide in membrane bilayers, which indicates that the peptide increases the probability of protrusion events^7,14,18,27^. It has also been shown that the peptide interacts with the lipid headgroups, mainly through the N-terminal group, which induces these headgroups to penetrate deeper into the membrane (headgroup intrusion)^7,14,20,27^. A recent study using multiscale simulations indicates that hemagglutinin-catalyzed membrane fusion is a two-stage process: first lipid-tail protrusion induced by the fusion peptide catalyzes stalk formation and second, the fusion peptide and transmembrane domain interact with the distal membrane leaflet leading to hemifusion diaphragm formation and fusion pore opening^27^. This study also shows that the protonation of the N-terminal site strongly influences the ability of the peptide to interact with the distal membrane leaflet and promote stalk widening^27^.

Although the detailed mechanism of the pH-induced conformational change of hemagglutinin has not been elucidated, it seems clear that it involves the weakening of the interaction between HA1 head region and the HA2 stalk, plus the weakening of the contacts between the fusion peptide and the surrounding residues. This effect ultimately leads to the conversion of the hemagglutinin structure into an extended conformation, in which the fusion peptide is located at the tip and inserts into the host membrane^3^. It is not clear if the pH drop affects the fusion peptide itself, although studies have shown that the isolated peptide is more active at low pH, indicating that pH does indeed affect the peptide's properties^5,6,8,28,29^.

In this work, we conducted a thorough analysis of the effect of pH on the influenza fusion peptide properties, by combining experimental data from biophysical techniques with results from constant-pH molecular dynamics (cpHMD) simulations. A Förster Resonance Energy Transfer (FRET)-based analysis revealed that the percentage of peptide-induced lipid mixing is twofold higher at pH 5 relative to pH 7.4. The cpHMD simulations elucidated the effect of pH on the peptide properties and allowed us to perform a detailed characterization of the peptide-membrane interaction and pinpoint the residues which are crucial for the peptide’s ability to interact with and perturb the host membrane.

## Materials and methods

### Materials

The peptide used in this study corresponds to the first 23 residues of the HA2 subunit of the influenza subtype H1, flanked by a serine residue (GLFGAIAGFIEGGWTGMIDGWYGS). This peptide is identical to the one that was studied by Lorieau et al.^10^, without the 6 residue-long hydrophilic tail that was added in their study. The peptide was purchased with purity higher than 95% from JPT Peptide Technologies GmbH (Berlin, Germany) and used directly as supplied from the manufacturer.

1-palmitoyl-2-oleoyl-sn-glycero-3-phosphocholine (POPC) and 1-palmitoyl-2-oleoyl-sn-glycero-3-phosphoethanolamine (POPE) were obtained from Avanti Polar Lipids (Alabaster, AL). Dipalmitoylphosphatidylehtanolamine-sulforhodamine B (RhB-PE) and 1,2-dihexadecanoyl-sn-glycero-3-phospho[N-4-nitrobenz- 2-oxa-1,3-diazolyl]ethanolamine (NBD-PE) were purchased from Sigma (St. Louis, MO). Acetate buffer (20 mM sodium acetate, 150 mM NaCl, pH 5) was used in the measurements performed at pH 5 and HEPES buffer (10 mM HEPES, 150 mM NaCl, pH 7.4) was used in the measurements performed at pH 7.4. Peptide stock solutions were prepared by dissolving the peptide in dimethylsulfoxide (DMSO) before dilution with the buffer. The solubilization of all peptides was improved with mild bath sonication. Fluorescence spectroscopy measurements were conducted at room temperature in a Varian Cary Eclipse fluorescence spectrophotometer (Mulgrave, Australia). All the experimental assays were conducted in triplicate.

### Membrane interaction studies

Membrane partition studies were carried out with large unilamellar vesicles composed of POPC/POPE 50:50 (mol%). Large unilamellar vesicles with ~ 100 nm diameter were obtained by extrusion techniques^30^. The studies were performed by adding small volumes of concentrated large unilamellar vesicles stock solutions to the peptide samples at 16 μM, with a 10 min incubation before measurements. Fluorescence emission spectra were scanned in the 300–450 nm range with an excitation wavelength of 280 nm. The fluorescence intensities were corrected for successive dilutions, background intensities and scatter. Partition curves were plotted, and the partition coefficient, *K*_*p*_, was determined as previously described^31^ to compare the membrane affinity of the peptide at different pH values.

### Lipid mixing

To study the fusion peptide’s ability to induce lipid mixing at different pH values, a Förster Resonance Energy Transfer (FRET)-based assay was used as previously described^32^. This assay is based on the decrease in resonance energy transfer between two membrane probes, RhB-PE and NBD-PE, when the lipids of the vesicles labeled with both probes are allowed to mix with lipids from unlabeled vesicles. The concentration of each of the fluorescent probes within the pre-fusion large unilamellar vesicles membrane was 0.6 mol%. For this assay large unilamellar vesicles composed of POPC/POPE 50:50 (mol%) was used and prepared as described above. Labeled and unlabeled vesicles in a proportion of 1:4 were used at a total final lipid concentration of 100 µM. The fluorescence was measured with excitation at 470 nm and emission recorded between 500 and 650 nm. Phospholipid mixing was quantified on a percentage basis:

$$\% {\text{Fusion efficiency}} = \left( {{\text{R}} - {\text{R}}_{0} } \right)/({\text{R}}_{{{1}00\% }} - {\text{R}}_{0} )$$

where R is the value of the ratio between the fluorescence intensity with emission at 530 nm and 588 nm, corresponding to the maximum fluorescence emission of NBD and RhB, respectively, obtained 10 min after the peptide’s addition (at a final concentration of 16 µM) to a mixture containing large unilamellar vesicles having 0.6 mol% of each probe plus large unilamellar vesicles without any fluorescent probe. R_0_ is the ratio before peptide addition (constant during the evaluated time range), and R_100%_ the ratio after addition of Triton X-100 at a final concentration of 1% (v/v).

### Secondary structure analysis by FTIR spectroscopy

Fourier-transform infrared (FTIR) spectroscopy was used to analyze the peptide secondary structure as described in a previous work^7^. Attenuated total reflection Fourier-transform infrared (ATR-FTIR) spectra were obtained on a Bruker Tensor27 Bio ATR II spectrophotometer (Ettlingen, Germany) equipped with a MCT detector (broad band 1200–420 cm^−1^, liquid N_2_ cooled) at a resolution of 4 cm^−1^. The spectrometer was continuously purged with dry air. The internal reflection element was a silicone (Si) ATR plate. Peptide samples (at 0.5 mg/mL) in the absence and presence of POPC large unilamellar vesicles (2 mg/mL) were prepared in buffer, spread on the Si plate and dried until solvent evaporation. POPC large unilamellar vesicles were prepared as described above. For each spectrum a total of 120 scans (900–4000 cm^−1^) were averaged. Background of the internal reflection element was collected and subtracted to the samples. The determination of protein secondary structures was performed by deconvolution of the curve-fitting of the amide I band with Lorentzian functions.

### Constant-pH molecular dynamics simulations

Two sets of constant-pH molecular dynamics (cpHMD) simulations of the fusion peptide in a 1,2-Dimyristoyl-sn-glycero-3-phosphocholine (DMPC) membrane were performed, starting from two different orientations of the peptide in the membrane, which were obtained in a previous study and correspond to the final states of the productive replicates 1 and 4 analyzed in that work^14^. In that study we analyzed the peptide’s orientation in the membrane using atomistic molecular dynamics (MD) simulations, in which the lipids and water molecules started randomly distributed in the simulation box and were allowed to spontaneously assemble. Using this approach, we observed that the membrane spontaneously assembled around the peptide in 5 out of 10 simulations (productive replicates). In 4 out of the 5 productive replicates the peptide adopted a membrane-spanning and nearly vertical conformation, whereas in 1 of the replicates it adopted a more horizontal configuration, occupying only one membrane leaflet. These two states did not interconvert in 500 ns of MD simulation, which indicates that they are separated by a high energy barrier. Subsequent simulation studies by another group supported the existence of these two states and predicted that the membrane-spanning configuration corresponds to the lowest free energy minimum, which is separated from the other state by a high energy barrier of over 20 kJ/mol^15,16^.

**Figure 1:**
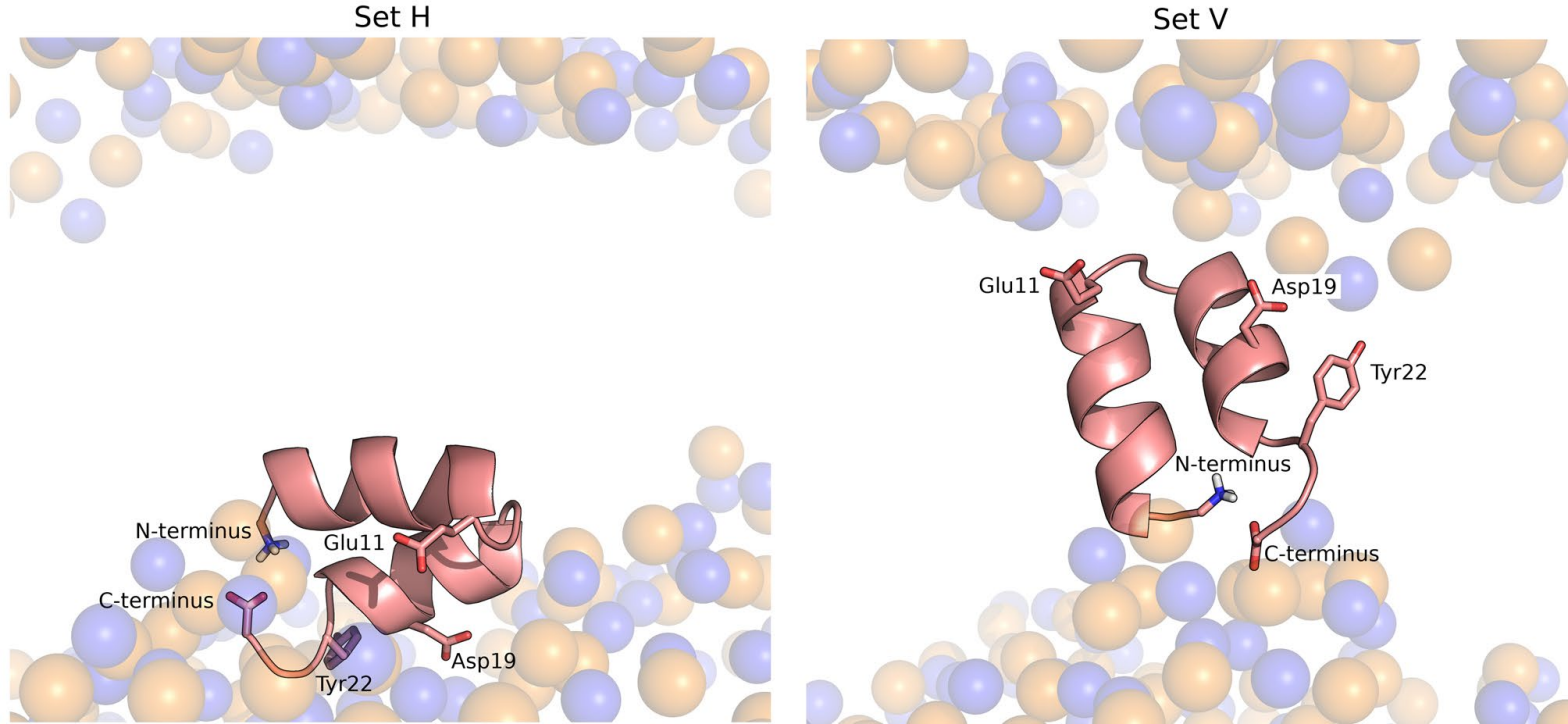
Starting configurations of the influenza fusion peptide in constant pH molecular dynamics simulations. These two configurations were obtained in a previous study^14^ and were used to initialize the two sets of simulations described in this work: set H (left) and set V (right). The molecular images of the two starting conformations were built with PyMOL^33^, using the final structures of replicates 1 (set V) and 4 (set H), obtained in a the previous study. The fusion peptide is shown using a cartoon representation colored in salmon and the lipid P and N atoms are depicted by transparent spheres colored in orange and blue, respectively. The titrable residues that were analyzed in this study are highlighted using sticks.

In the present work we analyzed the effect of pH on the two configurations adopted by the peptide in the membrane and used both of them as starting points of the cpHMD simulations. Thus, we setup two simulation sets, starting from the final states of the productive replicates 1 and 4 analyzed in the previous work^14^ These two sets of simulations are herein labelled set H (corresponding to a nearly horizontal starting orientation) and set V (corresponding to a nearly vertical starting orientation) (Fig. 1)—the PDB files of the starting configurations are available in supporting information and named start_H.pdb and start_v.pdb. Four different pH values were considered and five independent replicates were simulated at each pH value for each simulation set (a detailed description of the simulation setup is provided as Supporting Information). Given that fusion of the influenza membrane with the host membrane takes place at the endosome, we selected a pH range that includes the endosome pH (~ 5) and we also included pH 7 since we wanted to compare the peptide behavior in these two pH conditions. Additionally, we included the pH values 3 and 9 to analyze the effect of more extreme pH conditions and to be able to analyze the titration curves and predict the p*K*_a_ values of the relevant titrable residues present in this peptide.

The cpHMD method used in this work combines a stochastic titration methodology (based on continuum electrostatics and Monte Carlo calculations) with molecular dynamics (MD) simulations^34–37^. These calculations are run in a cycle where three block steps are sequentially repeated several times: (1) a Poisson–Boltzmann/Monte Carlo (PB/MC) block that assigns the protonation state of each residue in a given conformation, based on protonation free energy calculations performed with a continuum electrostatics method and Monte-Carlo simulations to sample protonation states; (2) a solvent relaxation block corresponding to a short MD simulation (0.1 ps in the present work), with all the system frozen except for the solvent, which enables the solvent molecules to adapt to the new protonation state; (3) an unconstrained MD simulation block (10 ps in the present work), which is used to sample new conformations with the protonation state assigned in step (1). The cpHMD simulations were run until the average protonation was reasonably converged, as discussed in the results section, which resulted in individual simulation lengths of 1 µs per replicate, with a combined simulation time that amounts to 40 µs.

### PB/MC settings

The MEAD package^38^ version 2.2.9 was used to calculate the PB-derived energy terms, using atomic charges and radii derived from the GROMOS 54A7 force field^39^, and the p*K*_a_ values of model compounds described in Table 1 of reference^40^. The molecular surface was computed by considering a rolling probe of radius 1.4 Å, and the Stern layer was 2 Å. The temperature and ionic strength were set to 310 K and 0.1 M, respectively, and the dielectric constants assigned to the molecular interior and the solvent were set to 2 and 80, respectively. A finite difference method was used to solve the PB linear equation using a two-step focusing procedure, using grid spacings of 1.0 and 0.25 Å, with a total of 81 grid points in each step. The software PETIT^41^ was used to sample the protonation states using 100,000 Monte Carlo steps in each calculation. At each step all individual sites and site pairs with coupling above 2.0 p*K*_a_ units were allowed to change to a random protonation state (including tautomeric forms)^41,42^ and a Metropolis criterion^43^ was used to accept or reject the new protonation state.

**Table 1:** p*K*_a_ values computed from constant pH MD simulations. The p*K*_a_ values and the respective errors were calculated as described in reference^54^. The Tyr22 pKa values are not defined in the pH range analyzed because this residue remained fully protonated at all pH values.

Site	Set H p*K*_a_	Set V p*K*_a_
N-ter	6.14 + − 0.53	7.24 + − 0.16
Glu11	7.63 + − 0.17	7.45 + − 0.13
Asp19	5.01 + − 0.18	6.97 + − 0.31
Tyr22	ND	ND
C-ter	3.95 + − 0.07	5.12 + − 0.16

### MM/MD settings

The molecular dynamics simulations were performed with the GROMACS^44^ package version 4.0.7^45^ with an integration timestep of 2 fs and using periodic boundary conditions. The GROMOS 54A7^39^ force field was used to model the peptide, the SPC model was used for water^46^ and the DMPC parameters used were the ones developed by Poger et al.^47,48^. A twin-range cutoff of 8/14 Å was used for nonbonded interactions, updating the neighbor list at every 5 simulation steps. Long-range electrostatic interactions were treated with the Generalized Reaction Field method^49^, setting the dielectric constant to 62^47,48^ and the ionic strength to 0.1 M. Temperature coupling was applied separately to the peptide, membrane and solvent atoms, using the v-rescale algorithm^50^, setting the reference temperature to 310 K and using a temperature coupling constant of 0.1 ps. The pressure was kept at 1 bar using the Parrinelo-Rahman coupling scheme^51^ applied independently in the XY and Z directions, with a coupling constant of 5 ps and an isothermal compressibility of 4.6 × 10^–5^ bar. All the bonds were constrained using the LINCS algorithm^52^, except for water molecules that were constrained using the SETTLE algorithm^53^.

## Results

### Partition coefficients of the influenza fusion peptide measured using fluorescence spectroscopy

Given that the influenza fusion peptide is intrinsically fluorescent due to the presence of tryptophan residues, fluorescence emission spectroscopy was used to study the interaction of the peptide with POPC/POPE large unilamellar vesicles at endosome mimetic pH (5.0) and at pH 7.4. The fluorescence quantum yield of the Trp residue is affected by the insertion of the peptides in the membrane and fluorescence emission intensity can, therefore, be used to estimate the peptide partition coefficient (*K*_p_) between the aqueous and lipid phases. The partition coefficients at pH 5 and 7.4 were determined to quantify the extent of the peptides incorporation in large unilamellar vesicles. The results obtained show that the peptide has a slightly higher affinity for POPC/POPE large unilamellar vesicles at pH 7.4 ((2.9 ± 0.4) × 10^4^) when compared to pH 5 ((1.0 ± 0.1) × 10^4^).

### Fusogenic activity of the influenza fusion peptide analyzed by a FRET-based assay

**Figure 2:**
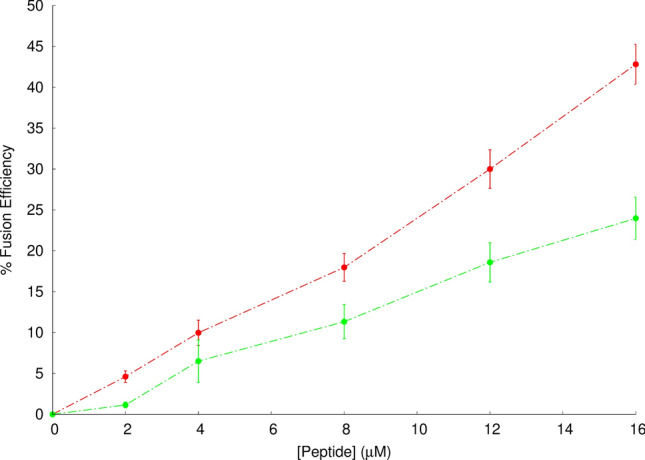
Spectral representation of efficiency of fusion in FRET-based assays in the presence of the influenza fusion peptide. To study the peptide’s ability to induce lipid mixing at pH 5 (red line) and pH 7.4 (green line), a Förster Resonance Energy Transfer (FRET)-based assay was used as previously described^32^. large unilamellar vesicles labeled with 0.6% mol of RhB-PE and NBD-PE were mixed with unlabeled liposomes to a final proportion of 1:4 and the energy transfer assay was evaluated in the presence of the fusion peptide after 10 min of incubation. The % fusion efficiency was calculated at different peptide concentrations as described in the Materials and Methods section. Each point corresponds to the average value of three independent replicates and bars represent the corresponding standard deviation. The data at pH 5.0 has been collected in a previous work^7^ and is shown here for comparison with the data obtained at pH 7.4.

In order to study the peptide’s fusogenic activity, a FRET-based assay was performed using POPC/POPE large unilamellar vesicles to determine the percentage of lipid mixing at pH 5.0 and 7.4. The curves displayed in Fig. 2 were used to calculate the percentage of lipid mixing as described in the “Materials and methods” section. The results show that the percentage of lipid mixing at pH 5 is 42.8 ± 1.7%, whereas at pH 7.4 the value drops to 23.9 ± 1.8%. Since the fusion peptide partition coefficient follows an inverse trend, the higher peptide fusion efficiency observed at pH 5 cannot be attributed to a higher affinity for the membrane. This indicates that, although the peptide interacts less with the lipid bilayer at pH 5 than at pH 7.4, it is more efficient in perturbing the membrane and inducing lipid mixing at the endosome mimetic pH.

### Influenza fusion peptide secondary structure analyzed by FTIR

**Figure 3:**
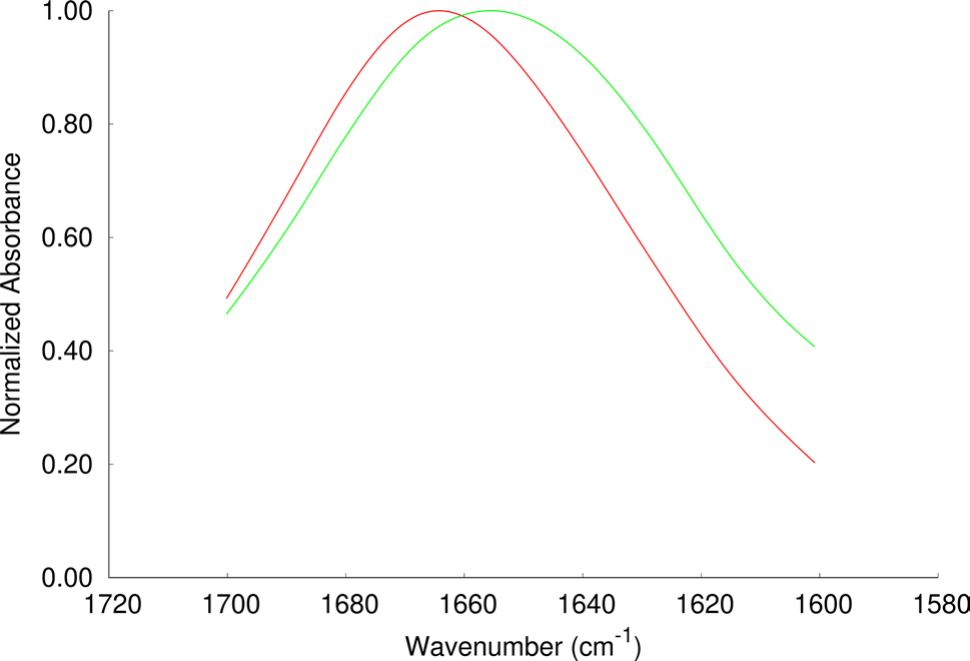
ATR-FTIR amide I band for the influenza fusion peptide in presence of POPC large unilamellar vesicles. Attenuated total reflection infrared (ATR-FTIR) spectra were obtained on a Bruker Tensor27 Bio ATR II spectrophotometer at a resolution of 4 cm^−1^ as described in the Materials and Methods section. All the spectra were normalized and the samples were prepared in pH 5.0 (red line) and pH7.4 (green line). The spectrum at pH 5.0 has been collected in a previous work^7^ and is shown here for comparison with the spectrum obtained at pH 7.4.

The secondary structure of the influenza fusion peptide was analyzed by ATR-FTIR spectroscopy. The spectra of the peptide in the presence of POPC large unilamellar vesicles were collected at pH 5.0 and 7.4 (Fig. 3). The wavenumber ranges of the amide I absorption bands indicate that the peptide adopts a predominantly α-helical conformation in both pH conditions. The peak obtained at pH 5 is centered at 1664 cm^−1^, whereas the peak obtained at 7.4 is centered at 1657 cm^−1^ and is broader.

### Temporal evolution of the fusion peptide properties in constant-pH molecular dynamics simulations

To shed light into the molecular details underlying the pH effect on the peptide’s structural properties and its interaction with the membrane, we performed constant-pH molecular dynamics simulations (CpHMD) simulations. Previously, we had observed that the fusion peptide can adopt two different configurations in the membrane: an interfacial conformation where the peptide has a parallel orientation relative to the membrane plane; and a membrane-spanning conformation where it adopts a nearly vertical orientation (Fig. 1)^14^. In our previous study, these two configurations did not interconvert, which indicates that they are separated by a high-energy barrier. Subsequent studies by another group have made similar observations and predicted that the membrane spanning configuration is the most probable configuration of the fusion peptide with a protonated N-terminal and that the energy barrier separating the two states is higher than 20 kJ/mol^15,16^. In the present work, we decided to perform cpHMD simulations starting from each one of these two configurations, which are labelled as set H (for the interfacial horizontal configuration) and set V (for the vertical membrane-spanning configuration). Given that fusion of the influenza plasma membrane with the host membrane takes place at the endosome, we selected a pH range that includes the endosome pH (~ 5) and we also included pH 7 since it corresponds to a neutral pH. Additionally, we included the pH values 3 and 9 to analyze the effect of more extreme pH conditions and to be able to analyze the titration curves and predict the pKa values of the relevant titrable residues present in this peptide. Five independent simulations of 1 μs were performed at each pH, for each starting configuration.

**Figure 4:**
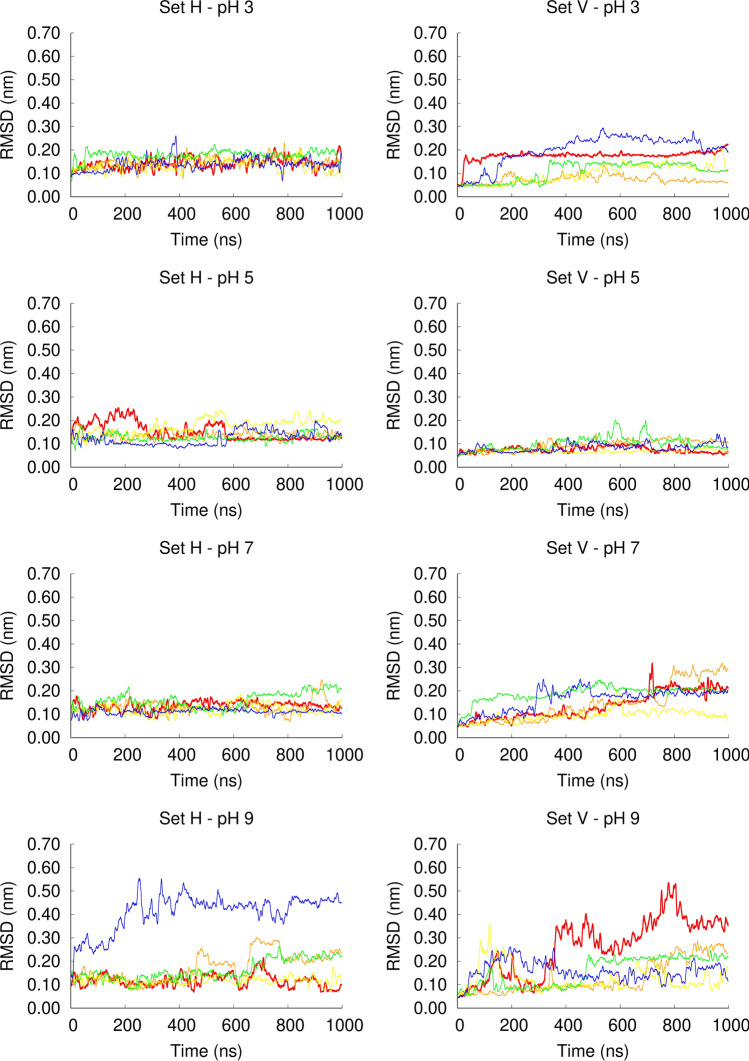
Temporal evolution of the Cα root mean square deviation in constant pH MD simulations. The root mean square deviation (RMSD) was calculated with the GROMACS *g_rmsd* tool, using the starting structure of each simulation as the reference structure, excluding the initialization steps from the calculation. Only the Cα atoms were used for fitting and for the RMSD calculations. The plots on the left and right columns correspond to the simulation sets H and V, respectively and each row corresponds to a given pH: pH 3 (1st row), pH 5 (2nd row), pH 7 (3rd row), pH 9 (4th row). Each plot line corresponds to an independent replicate simulation performed in the same conditions (red: replicate 1; orange: replicate 2; yellow: replicate 3; green: replicate 4 and blue: replicate 5).

The RMSD analysis (Fig. 4) shows that in the simulation set H the fusion peptide is stable between pH 3 and pH 7, whereas in one of the replicates at pH 9, the structure changed considerably and, visual inspection indicated that the C-terminal helix became unfolded. In the simulation set V, the peptide is very stable at pH 5 (corresponding to the endosome pH), in which the peptide was shown to be more active. At this pH value, all the replicates have low RMSD values. A larger heterogeneity among replicates is observed at the other pH values (3, 7 and 9) and the peptide is more unstable at pH 9, as observed for the simulation set H. Overall, these results indicate that the peptide is stable in the pH range 3–7, becoming more unstable at pH 9. However, we note that the peptide is still mainly helical, even at high pH, and the unfolding of the C-terminal helix was observed in only 2 out of 10 replicates at pH 9. These results are in line with the FTIR data showing that the peptide is helical at both pH 5 and 7.4 and that the spectra obtained at pH 7.4 is broader than the one obtained at pH 5, which indicates that at higher pH other types of structural elements might be present, including unstructured regions.

To analyze the peptide orientation in the membrane during the simulation, we measured the tilt angle between the N and C-terminal helices and the membrane plane (Figures S1 and S2 in Supporting Information). The plots show that in the simulation set H the peptide remains horizontal at all pH values, although the tilt angle slightly increases with pH. In the simulation set V, at low pH (3 and 5) the peptide maintains a high tilt angle throughout the simulations, whereas at pH 7 and 9, in some replicates, the tilt angle decreases during the simulation, which means that the peptide adopts a more horizontal orientation.

In cpHMD simulations, the protonation states of titrable residues can change during the simulation and the average proton occupancies at different pH values can be computed. Given that fusion of the influenza plasma membrane with the host membrane takes place at the endosome (pH ~ 5), we focused our analysis on the sites that may be titrable in the pH range 3–9, namely the N-terminal site of Gly1, the side-chain carboxyl groups of Glu11 and Asp19, the side-chain hydroxyl group of Tyr22 and the C-terminal site of the flanking residue Ser24.

The analysis of the average protonation over time (Figures S3 and S4 in Supporting Information) indicates that Tyr-22 remains fully protonated over all the pH values analyzed, in both simulation sets, indicating that this residue is not titrable in a biologically relevant pH range. Therefore, we could not compute the titration curve for this residue and it was not taken into account in subsequent analysis. All the other sites displayed different protonation profiles at different pH values and their titration curves and membrane-effect were further investigated.

### Residue titration curves computed from cpHMD simulations

**Figure 5:**
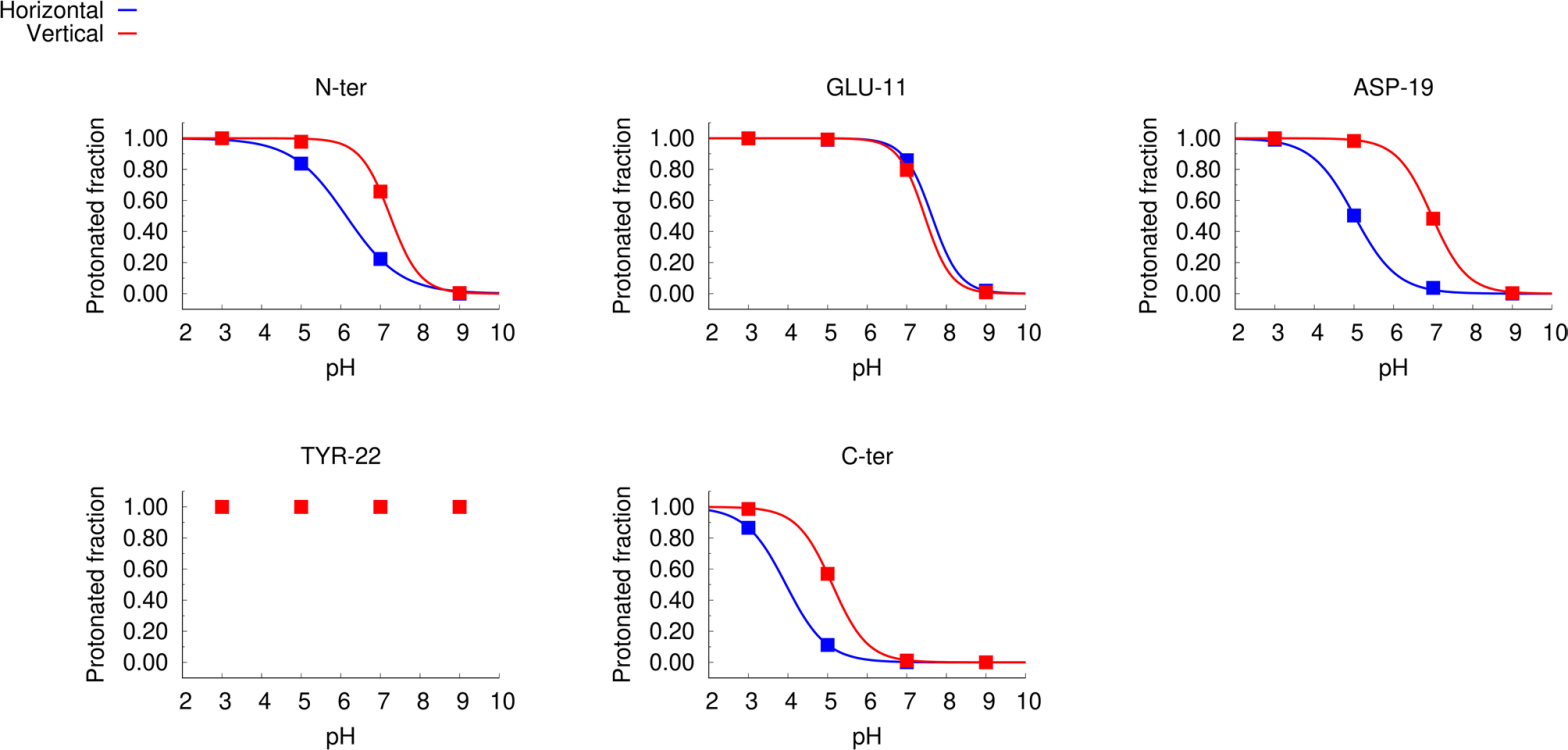
Titration curves computed from constant-pH MD simulations. The plots show the titration curves computed for each titrable group: N-terminal amine (N-ter), side chain carboxyl groups of Glu11 (Glu11) and Asp19 (Asp 19), side chain hydroxyl group of Tyr22 (Tyr22) and C-terminal carboxyl group of Ser24 (C-ter). The points represent average proton occupancies over the last 400 ns of simulation over all the replicates performed in each condition and the lines are the corresponding Hill curve fits. The blue and red colors represent the simulation sets H and V, respectively. The average protonation values obtained for each replicate at each pH value are shown in figure S5.

The average protonation values over the last 400 ns of simulation (Figure S5) were used to compute the titration curves of the titrable sites: N and C-terminus and the side chain carboxyl groups of Glu11 and Asp19 (Fig. 5). The analysis of the titration curves reveals that Glu11 displays similar protonation behaviors in the two sets of simulations (set H and set V), whereas differences can be observed in N-ter, C-ter and Asp19 protonation curves in the two simulation sets, which are more accentuated in the case os Asp19.

The titration curves of the N-terminal site show that the proton occupancy at neutral pH (7) is different from that observed at the endosome pH (~ 5)^55^, particularly in the simulation set H. Analyzing the N-terminus titration curves in more detail shows that in the simulation set H the protonation probabilities at pH values 3, 5 and 7 are 1, 0.84 and 0.22, respectively and in the simulation set V the protonation probabilities are 1, 1 and 0.65 for the same pH values. This indicates that at low pH the N-terminal site has a greater tendency to be protonated, particularly, when it adopts a horizontal conformation. The computed p*K*_a_ value of the N-terminal site in simulation sets H and V are 6.14 ± 0.53 and 7.24 ± 0.16, respectively (Table 1), which is lower than the value of 8.8 obtained in a previous NMR study^11^. However, we note that in that study the peptide was in a detergent micelle and had a lysine tail attached, which may influence the results.

The titration curves show that Glu11 is mostly protonated in the pH range between 5 and 7. On the other hand, the protonated fraction of Asp19 changes considerably in this pH range and the curves obtained for the simulations sets H and V are different. This residue is located near the center of the C-terminal helix, on the exterior face of the fusion peptide (Fig. 1). In the horizontal configuration, it is located in the headgroup region and exposed to the solvent, whereas in the vertical configuration it is buried inside the membrane (Fig. 1) and, thus, has a higher tendency to protonate. Glu11 and Asp19 have considerably higher p*K*_a_ values than the typical values for these amino acid residues in water (Table 1). This is due to the fact that, in the systems studied here, they are exposed to the hydrophobic membrane environment and, thus, have a higher tendency to protonate and become neutral. A previous NMR study has also shown that these residues have high p*K*_a_ values^11^, particularly in the case of Glu11, although the values obtained in that study (5.31 and 4.35 for Glu11 and Asp19, respectively) are lower than the ones computed in the present work. As explained above, these differences can be due to the fact that the peptide tested by NMR contained a lysine-tail (to make it more soluble) and the experiments were done in dodecylphosphocholine micelles. Overall, this analysis indicates that the protonated fractions of the N-terminal and the Asp19 sites change considerably between pH 5 and 7, which can be linked to the activity difference observed in this pH range, in our FRET-based analysis.

As mentioned above, Tyr22 remains fully protonated in the pH range analyzed and could not be titrated. Regarding the C-terminal site, the estimated p*K*_a_ values for the simulation sets H and V are 3.95 + − 0.07 and 5.12 + − 0.16, respectively. These results are representative of the model peptide that was used in the experimental analysis presented in this manuscript. However, we note that, during the infection process, the fusion peptide is attached to the rest of the hemagglutinin protein through the C-terminal side and is not titrable. For this reason, the protonation profile of the C-terminus site shown in Fig. 5 cannot be extrapolated to a biological context.

### Effect of pH on the fusion peptide structural properties: the vertical conformation is more stable at low pH

**Figure 6:**
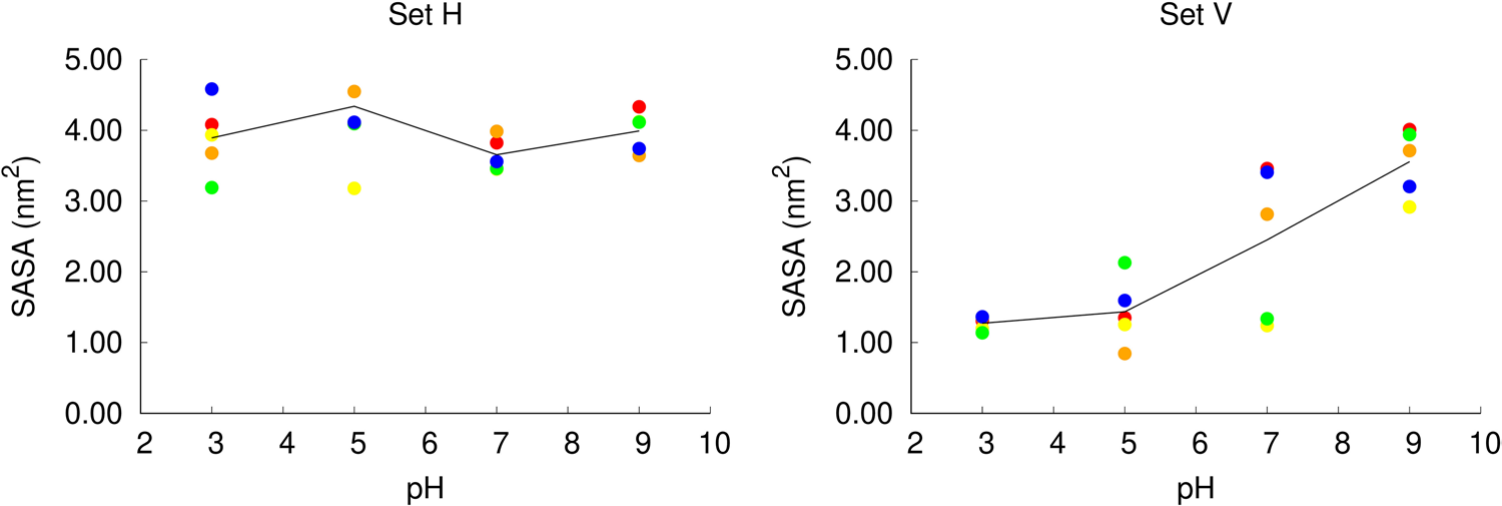
Average solvent accessible surface area of the fusion peptide in constant-pH MD simulations. The fusion peptide solvent accessible surface area (SASA) was computed with the GROMACS g_sas tool, which implements the algorithm described by Eisenhaber et al.^56^ The average SASA value for each replicate at each pH value was calculated over the last 400 ns of simulation for the simulation set H (left side) and V (right side). Each point corresponds to an independent replicate simulation performed in a given pH value (the color codes used are describe in Fig. 4) and the black line passes through the average value computed over all the replicates simulated at a given pH value.

Given that there is a close link between protonation and solvent exposure, we analyzed the solvent accessible surface area (SASA) of the peptide in different pH conditions, in the two sets of simulations (Fig. 6). In the simulation set H, the peptide is located just below the head groups, with one face exposed to the solvent. Our analysis shows that in these simulations the peptide has a large solvent accessible surface, which does not change considerably with pH.

Interestingly, in the simulation set V, the peptide’s solvent accessible surface area changes considerably with pH. At low pH (3 and 5), the peptide has a considerably lower solvent accessible surface area relative to the simulation set H, since it remains deeply inserted in the membrane and shielded from the solvent throughout the simulations. At pH 7, the replicates have a heterogeneous behavior: three replicates have a high solvent accessible surface area and two replicates have a lower solvent accessible surface area. At pH 9, the peptide has a high solvent accessible surface area in all replicates, in the same range observed in the simulation set H. To gain further insight into this matter, we built scatter plots of the average solvent accessible surface area vs average protonation of each residue for the simulation sets H and V (Figure S6 in Supporting Information). The results show that in the simulation set H there is no apparent correlation between these two properties. In the simulation set V, on the other hand, a correlation between the two properties is observed, in particular, for the Asp19 residue. This can be explained by the fact that at high pH values Asp19 becomes deprotonated and charged and has a higher tendency to interact with water molecules.

Analyzing the water density across the membrane (Figure S7 in Supporting Information), we can see that in the simulation set H there is no water in the center of the membrane, as is usually the case in membrane simulations. However, in the simulation set V, the presence of water molecules in the bilayer center depends on pH: at low pH the water density is approximately zero, whereas at high pH the average water density increases.

**Figure 7:**
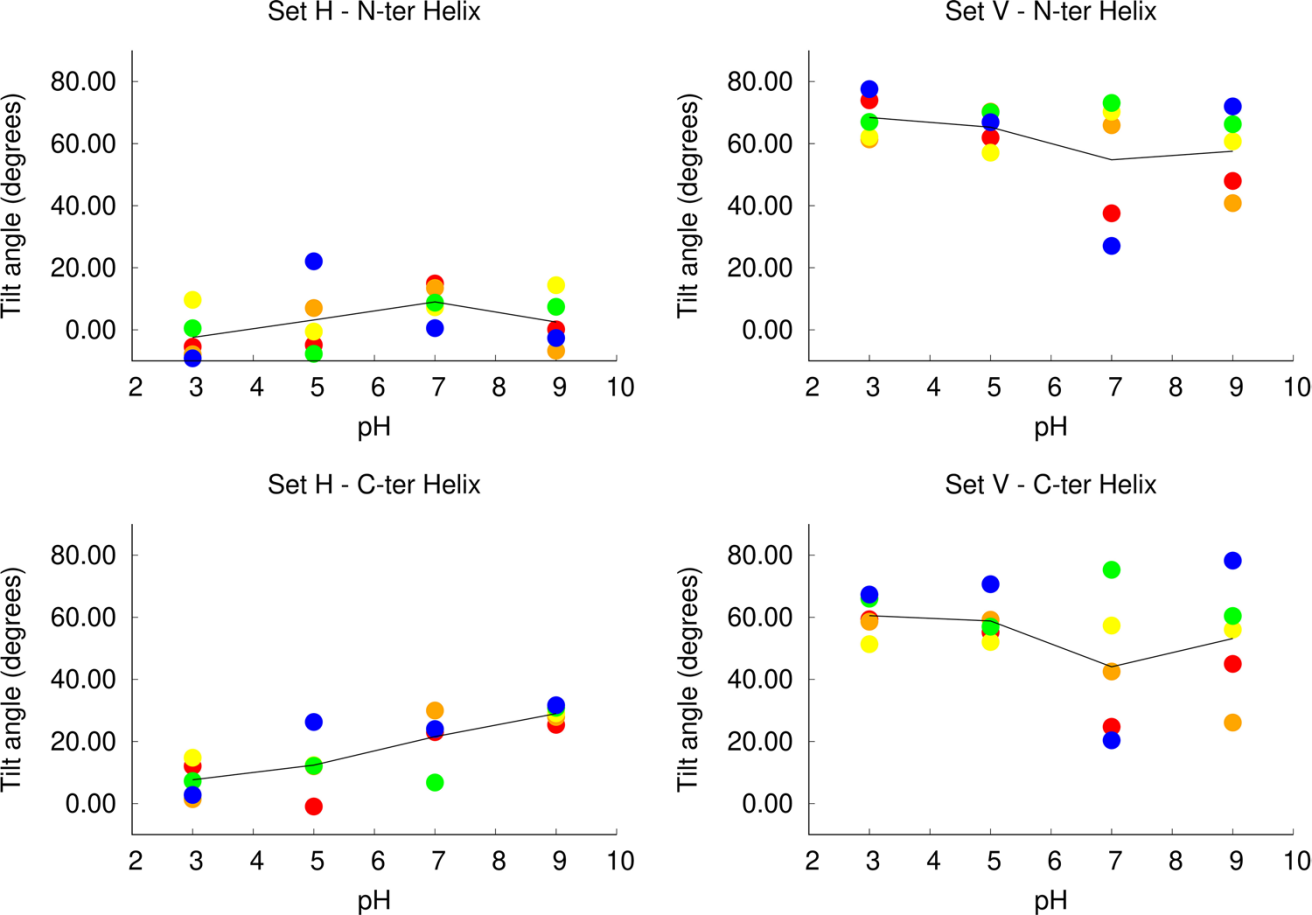
Average tilt angle of the fusion peptide in constant-pH MD simulations. The tilt angles for the N and C-terminal helices were computed as described in Figures S1 and S2 (Supporting Information), respectively, and averaged over the last 400 ns of simulation. The plots correspond to the tilt angle of each of these helices in the two simulation sets: N-terminal helix in simulation set H (top-left); N-terminal helix in simulation set V (top-right); C-terminal helix in simulation set H (bottom-left); C-terminal helix in simulation set V (bottom-right). Each point corresponds to an independent replicate simulation performed at a given pH value (the color codes used are described in Fig. 4) and the black line passes through the average value over all the replicated at a given pH. The C-terminal helix tilt angle of the replicates in which this helix unfolded at pH 9 was not calculated (replicates 5 in set H and 1 in set V).

**Figure 8:**
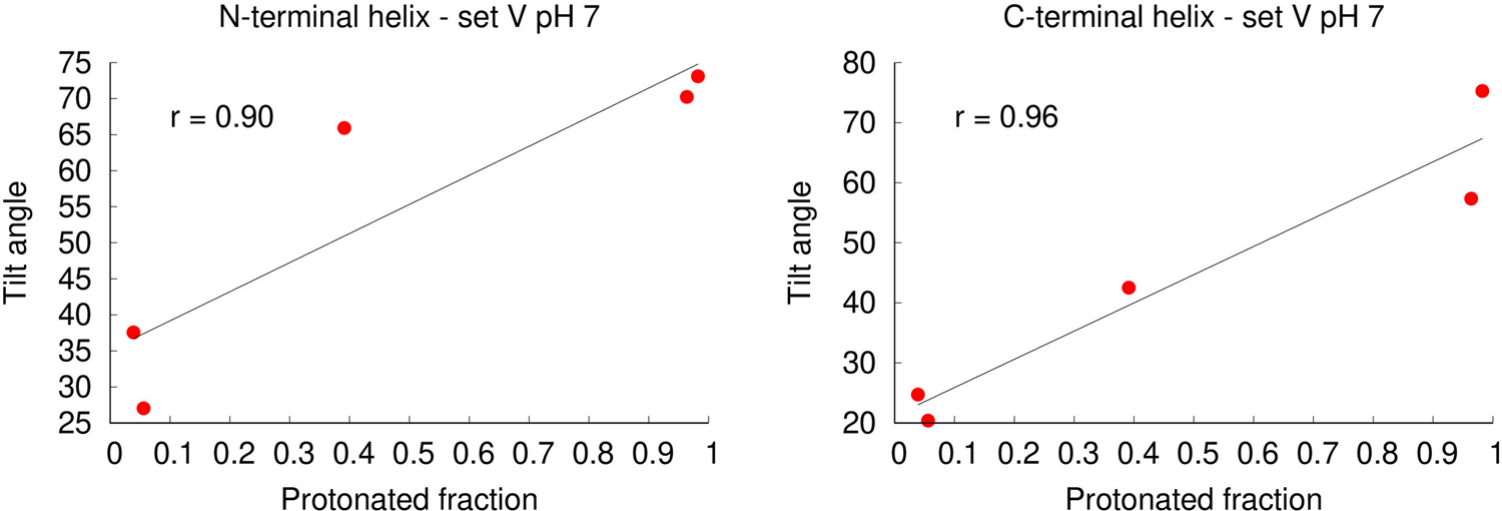
Scatter plots of the helix tilt angles vs average protonated fraction of Asp19 for the simulation set V at pH 7. The plots display the tilt angle as a function of the protonated fraction for the N-terminal (left side) and C-terminal (right side) fusion peptide helices. The average protonated fraction and the average tilt angle (computed as described in Figures S1 and S2) were calculated over the last 400 ns of simulation for each replicate of the simulation set V at pH 7. Each point represents one independent replicate simulation, the black line corresponds to the best linear fit to the points computed with gnuplot, and the r value indicates the statistical correlation.

To examine how pH affects the orientation of the peptide in the membrane, we measured the average tilt angles of the two peptide helices (labelled N-terminal and C-terminal helix, respectively), after equilibration (Fig. 7). In the simulation set H, the N-terminal helix displays a low tilt angle relative to the membrane plane (corresponding to a horizontal orientation) at all pH values. The C-terminal helix also displays a low tilt angle at all pH values, although it becomes slightly more tilted as the pH increases. In the simulation set V, we observe that the orientation of the peptide in the membrane is strongly affected by pH. At low pH values, both the N-terminal and the C-terminal helices display a high tilt angle in the membrane, compatible with a nearly vertical conformation, while at higher pH several replicates display lower tilt angles relative to the membrane plane. This behavior is clearly shown in the plots of the C-terminal helix. At pH 3 and 5 this helix adopts a large tilt angle in all the replicates, whereas in the simulations at pH 7 and 9 more than half of the replicates become considerably less tilted, having average tilt angles comparable with those observed in the simulation set H. These results indicate that as the pH increases the peptide tends to deviate from the vertical conformation (a movie illustrating this behavior is available in the supporting information – movie S1). Interestingly, there is a correlation between the average protonation of Asp19 and the tilt angle adopted by the peptide at pH 7, i.e. the replicates in which Asp19 is frequently deprotonated adopt more horizontal orientations (Fig. 8). Since this residue is located in the middle of the C-terminal helix, it stands close to the bilayer center when the peptide adopts a vertical orientation (Fig. 1). At high pH, Asp19 is frequently deprotonated becoming negatively charged, which makes the vertical orientation, where this residue is exposed to the membrane interior, more unstable.

To further test the hypothesis that the peptide tends to deviate from the vertical conformation at high pH, we performed 5 independent standard MD simulations with fixed protonation states, with all the titrable residues deprotonated, starting from the final state of a vertical CpH simulation (a detailed description is available in supporting information). We observed that in 3 out of 5 standard MD simulations (replicates 1, 2 and 4) the peptide adopted a horizontal conformation (Figure S8) and was interacting with only one membrane leaflet. The C-terminal helix (where the charged E11 and D19 residues are located) lied parallel to the membrane plane in the head group region and the N-terminal helix was slightly tilted and more deeply inserted inside the membrane. In replicates 3 and 5 the peptide did not lose all the contacts with the opposite leaflet. , the N- terminal end of the peptide tends to maintain the interaction with the lipid headgroups, and, as it moves from one side of the membrane to the other, it drags these headgroups to the center of the bilayer. This creates what seems to be a metastable state, in which the membrane is deformed with contacts between the two leaflets. We observed that in 2 of the replicates the membrane leaflets detached from one another after a few microseconds of simulation (replicates 1 and 2), whereas in replicates 3 and 5 other replicates, they did not and replicate 4 represents an intermediate state. Replicates 3, 4 and 5 seem to be trapped in a metastable state, in which the membrane is deformed. However, visual inspection of these trajectories indicates that they are heading in the same direction as replicates 1 and 2, although the membrane leaflets did not completely detach. Overall, these observations support the hypothesis that at high pH the fusion peptide tends to deviate from the vertical state and adopt a more horizontal and solvent-exposed conformation, although this is a slow process, with metastable states along the way.

Taken together these analysis highlight the importance of pH on the peptide orientation. At low pH, both the horizontal and the vertical conformations are stable and, at this point, we cannot determine which of the two states corresponds to the global minimum, since they are separated by a high barrier and do not interconvert. On the other hand, at high pH, the vertical conformation is less stable and the peptide tends to become less tilted, even when it starts from a vertical state. This can have important functional implications, since it can affect the peptide-membrane interaction profile and effect on the membrane properties. In fact, previous MD simulations have shown that the fusion peptide has a more pronounced effect on the membrane when it adopts a vertical, membrane-spanning conformation^14–16^.

### Fusion peptide interaction with the membrane: pH affects the interaction with the N-terminus

**Figure 9:**
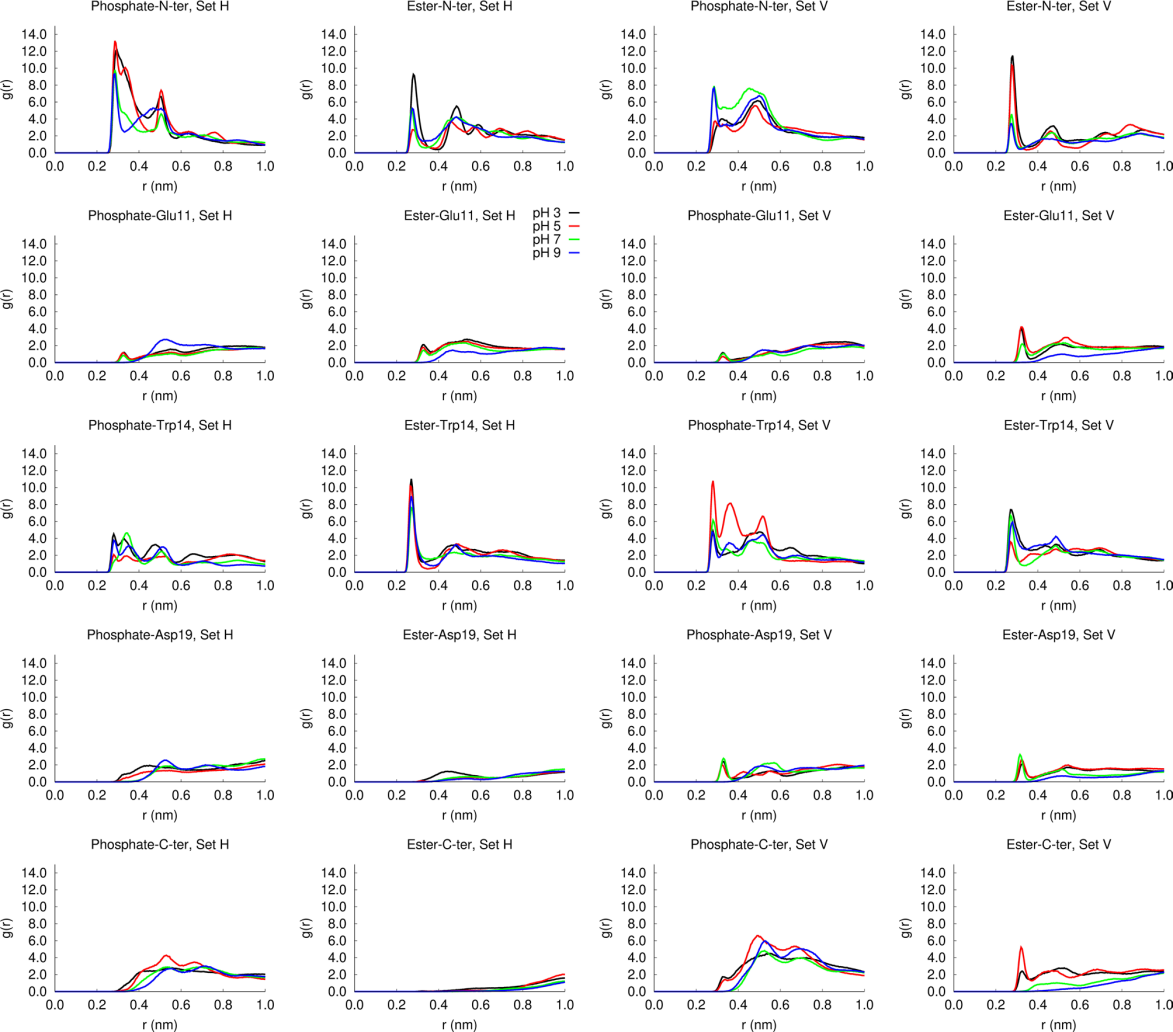
Radial distribution functions of the lipid phosphate and ester oxygens. Each row corresponds to a given residue: N-ter (1st row); Glu11 (2nd row); Asp19 (3rd row); C-ter (4th row). The 1st and 2nd columns correspond the radial distribution functions of the phosphate and ester oxygens in the simulation set H, respectively and the 3rd and 4th columns correspond the radial distribution functions of the phosphate and ester oxygens in the simulation set V, respectively. The trajectories of the last 400 ns of all the replicates performed at a given pH in each simulation set were concatenated and used to calculate the RDF with the GROMACS *gmx rdf* tool, using a reference atom for each residue: N-ter (main chain N); Glu11 (CG); Trp14 (NE); Asp19 (CD); C-ter (main chain C).

To investigate how pH affects the peptide interaction with the membrane, we computed the radial distribution functions of each of the residues of interest around the phosphate and ester oxygens (Fig. 9). This analysis shows that in the simulation set H, the peptide frequently interacts with the phosphate oxygens and this interaction is mediated mainly by the N-terminal site. Comparing the radial distribution functions obtained at different pH values, we observe larger peaks at pH 3 and 5, corresponding to acidic pH. We also observe that, in the simulation set H, Trp14 often interacts with the ester oxygens and the N-ter also interacts with the ester oxygens at pH 3. In the simulation set V, the N-terminal group frequently interacts with the ester oxygens, particularly at low pH values. This can be attributed to the fact that the N-terminus is more frequently protonated at low pH (as shown in the titration curves) and also to the higher stability of the vertical membrane-spanning conformation in acidic pH, which enables the N-terminal to penetrate deeper into the membrane. These results highlight the relevance of the N-terminal and Trp14 groups for the interaction of the influenza fusion peptide with the membrane.

**Figure 10:**
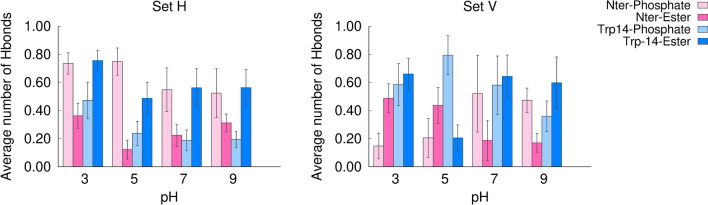
Average number of hydrogen bonds between the N-terminus and Trp14 with lipid phosphate and ester groups. The bar plots show the average number of hydrogen bonds per frame for the simulation sets H (left side) and V (right side). The number of hydrogen bonds in each frame of the final 400 ns of each replicate simulation was calculated with the GROMACS tool *gmx hbond*. The average number of hydrogen bonds per frame was then calculated for each replicate and the average over all the replicates was computed and is displayed in the bar plots. The error bars were calculated using a bootstrap method: for each condition, five new values were resampled with replacement from the original set of five replicates and their mean value was computed, this process was repeated 1000 times and the error was calculated as the standard deviation of these 1000 mean values.

To gain further insight into the nature of the interactions between the N-terminal and the Trp-14 residues with the lipids, we calculated the average number of hydrogen bonds between these groups, at each pH value, for both simulation sets (Fig. 10). The results show that the N-terminal and the Trp14 residues form frequent hydrogen bonds with the phosphate and ester groups, displaying different patterns in the two simulation sets. In the simulation set H, the N-terminus tends to form hydrogen bonds with the phosphate groups, which are more frequent at low pH, when this site is more often protonated. In this simulation set, Trp14 frequently interacts with the ester groups, particularly at pH 3.

**Figure 11:**
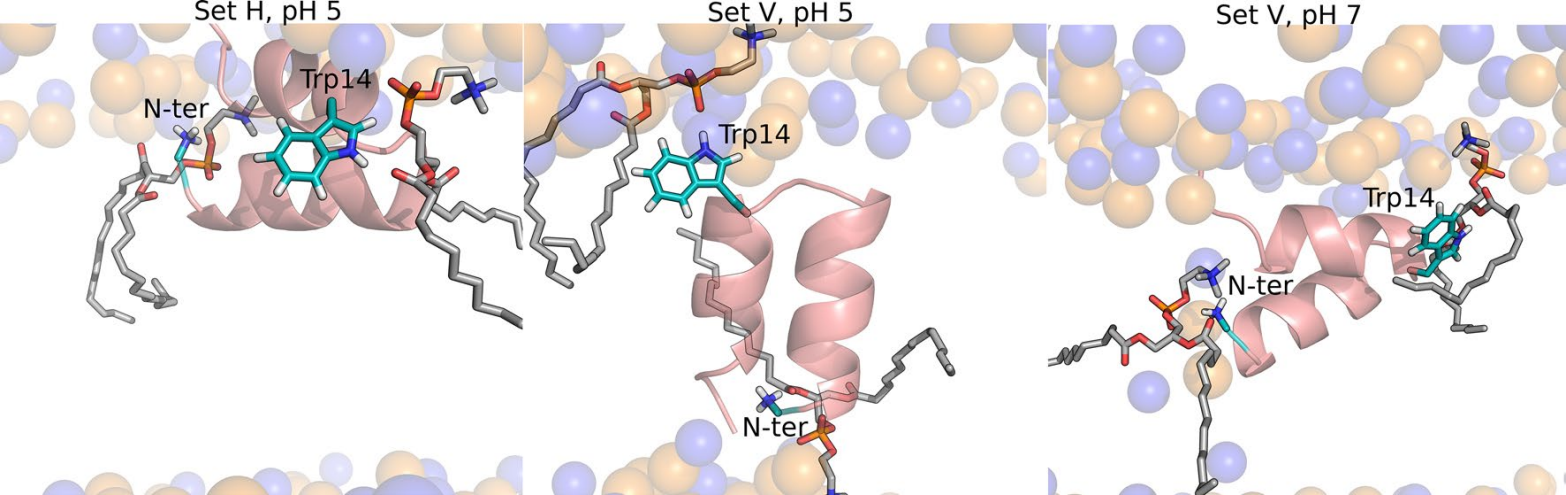
Illustration of the interaction between the N-terminal and Trp14 with the lipid phosphate and ester groups. The molecular images were built with PyMOL^33^ and correspond to the structures obtained after 805 ns in replicate 2 of the simulation set H at pH 5 (left side), after 620 ns in replicate 1 of the simulation set V at pH 5 (middle) and after 864 ns in replicate 5 of the simulation set V at pH 7 (right side). In the simulation set H the peptide interacts predominantly with the phosphate oxygens and Trp14 tends to form hydrogen bonds with the ester groups. In the simulation set V the hydrogen bonding pattern depends on pH: at pH 5 the N-terminus and Trp14 interact predominantly with the ester and phosphate oxygens, respectively, whereas at pH 7 the pattern is reversed. The peptide is represented as in Fig. 3, with the N-terminal and Trp14 residues depicted using sticks with cyan carbons. The lipids interacting with these residues are represented using sticks, with C, N, P and O atoms colored in gray, blue, orange and red, respectively.

In the simulation set V, the peptide has distinct hydrogen bonding patterns at low and high pH. At pH 5 (corresponding to the fusion pH), in which the vertical conformation remains stable, the N-terminal residue forms frequent hydrogen bonds with the ester groups, whereas Trp14 interacts mainly with the phosphate groups. On the other hand, at high pH the pattern is reversed, i.e., the N-terminus tends to form hydrogen bonds with the phosphate groups and Trp14 interacts more frequently with the ester groups (simulation snapshots illustrating the interaction of the N-terminus and Trp14 with the lipid groups are shown in Fig. 11). At pH 7 and 9 the pattern found in the simulation set V is more similar to the pattern found in the simulation set H, due to the fact that, in some replicates, the peptide tends to deviate from the vertical conformation and become more horizontal (Fig. 7).

Taken together with the analysis of the residue protonation and the orientation of the peptide in the membrane, our results indicate that pH affects the peptide orientation in the membrane, which in turn affects the pattern of hydrogen bonds with the lipid phosphate and ester groups. At pH 5, the vertical conformation is stable and this allows the N-terminus to penetrate more deeply into the membrane and establish hydrogen bonds with the ester groups, whereas at higher pH this conformation is less stable, which leads to less frequent N-terminus-ester hydrogen bonds.

### Pinpointing key residues for the fusion peptide activity: the importance of the N-terminal site

**Figure 12:**
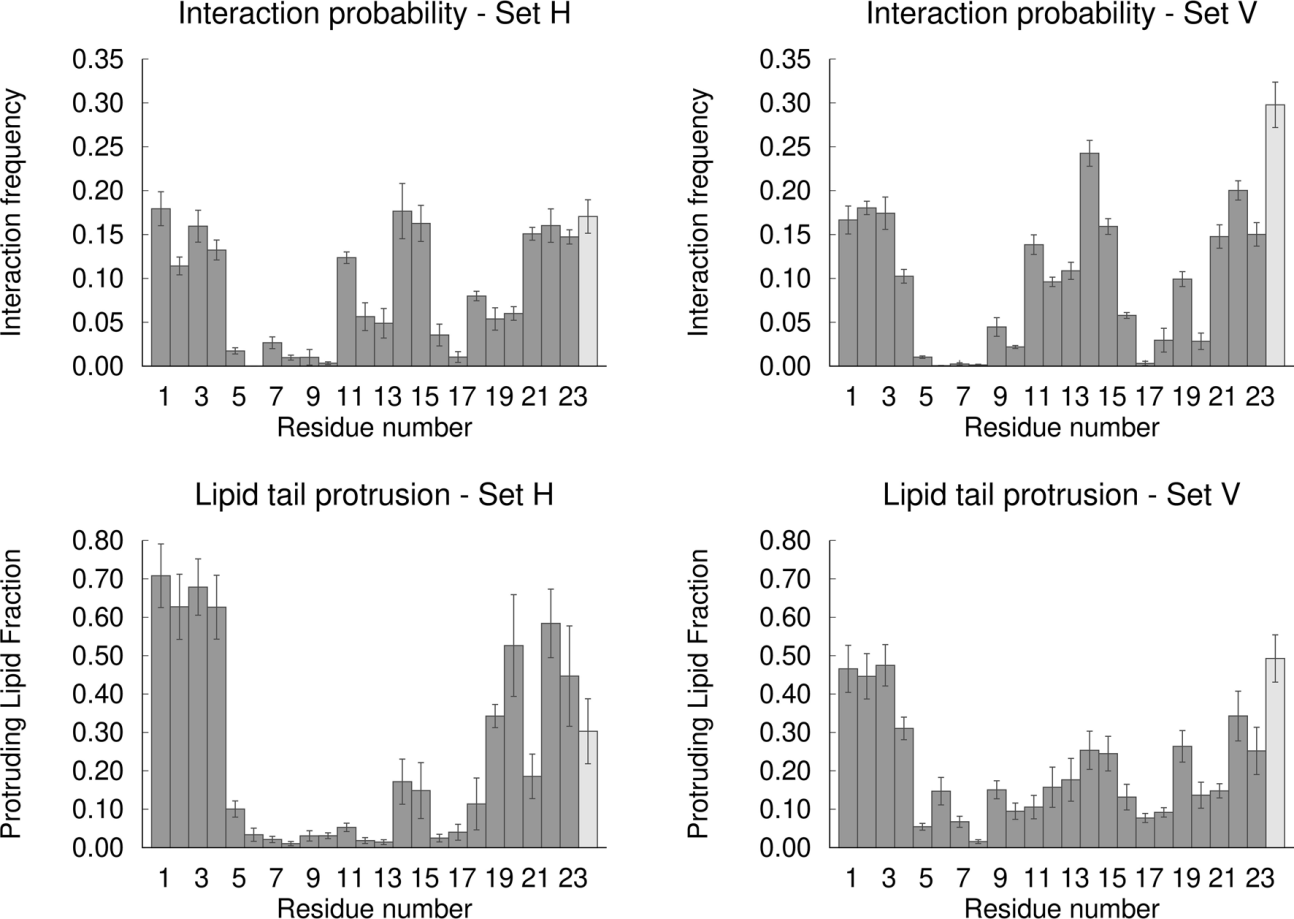
Interaction with DMPC oxygen atoms (top row) and lipid tail protrusion (bottom row) per peptide residue, for the set H (left column) and set V (right column) simulations at pH 5. The plots in the top row show the frequency of interaction of each residue with the lipid phosphate and ester oxygens. A minimum distance below or equal to 0.4 nm between a given residue and a lipid oxygen is used as interaction criteria and the interaction frequency was calculated by dividing the number of frames in which the residue is interacting with a lipid by the total number of frames. The plots in the bottom row show the fraction of lipids that are interacting with a given residue among the lipids that undergo lipid tail protrusion. A lipid is considered to be protruding if any of its tail carbons extend at least 0.1 nm in the z direction beyond the phosphate group. The protruding lipid fraction for each residue was calculated by dividing the number of protruding lipids that are interacting with that residue by the total number of protruding lipids in each replicate. The interaction and protrusion values correspond to the average obtained across all replicates and the error bars were calculated using a bootstrap method: for each residue, five new values were resampled with replacement from the original set of five replicates and their mean value was computed, this process was repeated 1000 times and the error was calculated as the standard deviation of these 1000 mean values. The C-terminal residue Ser24 is displayed in light grey to highlight the fact that this residue corresponds to a flanking residue and is not part of the fusion peptide itself.

Although several simulation and experimental studies have tried to elucidate the molecular mechanism by which the influenza fusion peptide promotes fusion, some of the details of this mechanism remain elusive. cpHMD simulations are particularly suited to shed light into this matter, since they allow proton binding/unbinding during the simulation, introducing a high level of realism that cannot be obtained with standard MD simulations. To investigate how the peptide interacts with the membrane and affects its properties, we focused on the simulations performed at pH 5, in which fusion occurs in biological conditions, and where the peptide has been experimentally shown to have a higher lipid mixing ability. As mentioned above, the C-ter residue Ser-24 was used here as a flanking residue and is not part of the actual fusion peptide. Moreover, in our model peptide this residue is free and can protonate/deprotonate, which does not happen in a biological context, since the C-terminal is attached to the rest of HA. Therefore, the results obtained for this residue cannot be directly extrapolated to the situation observed in the fusion context. For that reason, in the plots shown in Fig. 12 display the flanking Ser24 residue in light grey, whereas the residues that belong to the actual fusion peptide are displayed in dark grey.

The analysis of the frequency of interaction of each residue with the lipid phosphate and ester oxygens shows that this interaction occurs mainly through the residues located in the terminal and turn regions, with W14 displaying the largest number of contacts among the fusion peptide residues (Fig. 12—top row). The C-ter flanking residue Ser24 also has frequent interactions with the lipid oxygens, particularly in simulation set V. However, we note once again that this residue is not part of the actual fusion peptide and has different properties in this model peptide relative to its biological counterpart, which means that in a biological context the situation may be different.Interestingly, the contact profiles obtained for the simulation sets H and V are similar, showing that the peptide can form similar contacts with the lipid head groups in the two orientations. These contacts occur mainly through hydrophilic groups (carboxyl of E11, hydroxyl of T15 and Y22) and also through the side chain indole of two Trp residues (W14 and W21), as well as through the N-terminal group of G1 and the main chain NH groups of nearby residues (L2, F3 and G4). This interaction pattern is a consequence of structural properties of the influenza fusion peptide, which adopts a helix-turn-helix structure in lipid membranes, with most hydrophilic residues located in the terminals and close to the turn. Several studies have shown that this structure is very stable and that mutations that affect its stability lead to a decrease or loss of activity^7^. These results prompt us to hypothesize that the influenza fusion peptide (which has a very conserved sequence across viral subtypes) has evolved to adopt a very stable structure in lipid membranes, with an architecture that enables specific interactions with the membrane headgroups.

Previous simulation studies indicate that the occurrence of lipid tail protrusion lowers the energy barrier for membrane fusion^19^. Protrusion occurs when a carbon from a lipid tail projects beyond the corresponding lipid phosphate group^19^. Simulations of the influenza fusion peptide in the presence of membrane bilayers show that the probability of lipid tail protrusion is higher for the lipids that are in contact with the peptide, which suggests that this may be one of the mechanisms by which this peptide promotes membrane fusion^14,18^. One relevant question, which has not been yet addressed, is which residues play the most important roles in promoting lipid tail protrusion. To answer this question, we analyzed the fraction of lipids that are interacting with each residue among the protruding lipids (Fig. 12—bottom row).

The results show that the highest protruding lipid fraction is observed for lipids that are interacting with the residues located in the N-terminal region, followed by the residues on the C-terminal end of the fusion peptide, both in the horizontal and vertical simulations. The C-terminal flanking residue also contributes significantly to lipid tail protrusion. Nevertheless, this situation may be different in the context of the complete HA2 protein, where the C-terminal is attached to the rest of the protein. Curiously, W14, which has the largest number of contacts with lipid oxygens, and the other turn residues, do not contribute considerably to lipid tail protrusion, especially in the simulation set H. This indicates that W14 is important to stabilize the interactions of the peptide with the lipid membrane (which is in line with previous studies highlighting the role of Trp residues in membrane-interacting peptides^13^), but not as much to induce lipid tail protrusion.

**Figure 13:**
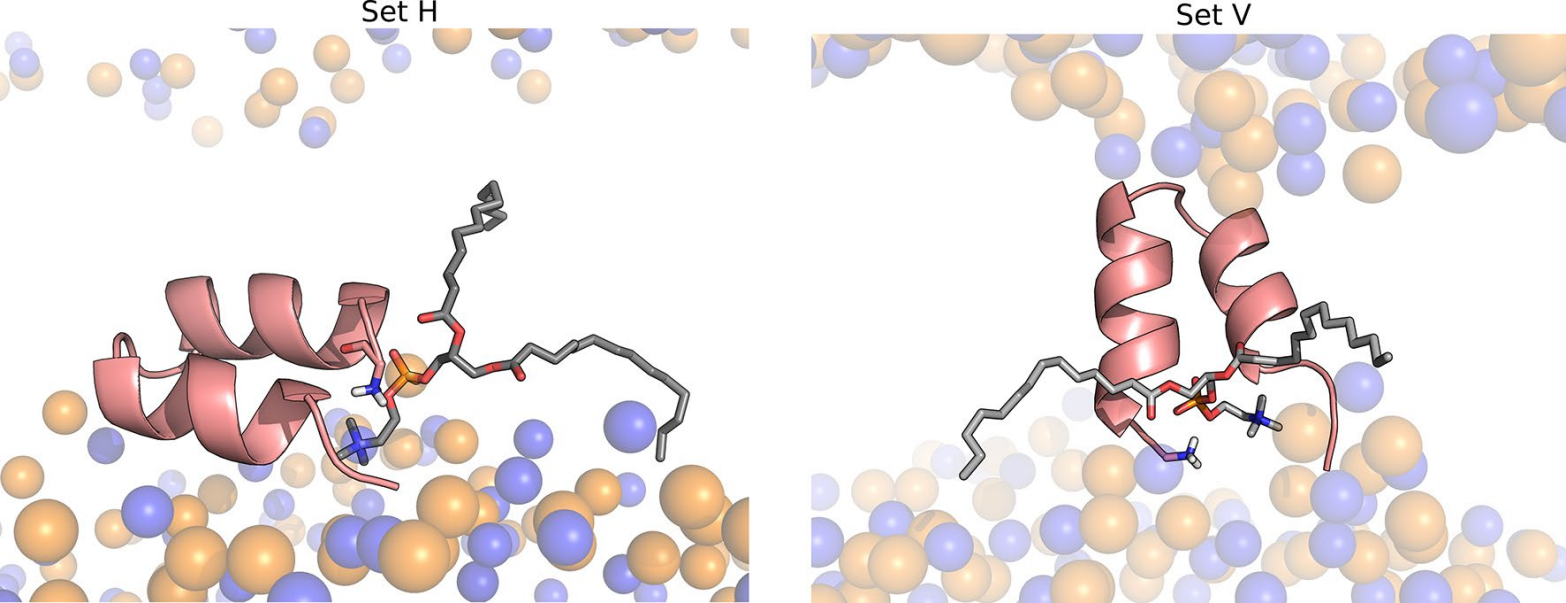
Illustration of lipid tail protrusion events upon interaction with the N-terminal region. The molecular images were built with PyMOL^33^ and correspond to the structures obtained after 114 ns in replicate 4 of the simulation set H at pH 5 (left side) and after 597 ns in replicate 4 of the simulation set V at pH 5 (right side). The peptide and lipid representation is identical to the one used in Fig. 3.

The peptide N-terminal region has the appropriate features to form strong contacts with the lipid oxygens. These interactions are mediated by the G1-Nter (which is nearly always protonated and positively charged at pH 5) and also by the main-chain NH groups of L2, F3 and G4. These residues can form a network of interactions with the lipid oxygens, although we note that these interactions are dynamic and not all of them are usually present at the same time. Additionally, the helical hairpin architecture adopted by the influenza fusion peptide allows the terminal ends of the two helices to work as “tweezers”, locking the oxygens in the middle. A lipid that interacts with and is pulled by these residues tends to become less ordered, which potentiates lipid tail protrusion that has been previously shown to be significantly increased in the presence of the influenza fusion peptide^14,18,19^ (simulation snapshots illustrating this behavior are shown in Fig. 13). We also observed that the phosphate atoms of the lipids that interact with the N-terminal region tend to become more buried in the membrane (we called this phenomenon lipid head intrusion, according to a previous reference^20^), which locally decreases the membrane thickness (Figure S9 in Supporting Information). These observations are in line with a recent study using coarse-grained MD simulations combined with the string method, which found that neuronal SNARE fusion proteins lower the barrier for the stalk formation by thinning the membrane^57^.

## Discussion

Using a combination of spectroscopic methods and constant-pH MD simulations we characterized the effect of pH on the activity and molecular properties of the influenza fusion peptide. The experimental analysis showed that the peptide is mostly helical in both pH values tested (5 and 7.4) and that the peptide’s ability to induce lipid mixing is ~ twofold higher at pH 5 (which mimics the endosome conditions) when compared to pH 7.4. This improved ability in mixing lipids is not accounted for by a membrane higher affinity at pH 5, since the lipid/water partition coefficient is higher at pH 7. The explanation resides on differential intrinsic properties of the peptides at pH 5 and pH 7.4.

Constant-pH MD simulations were herein applied for the first time to the study of viral fusion peptides. The high level of detail and realistic treatment of the coupling between structural properties and protonation, inherent to these simulations, allowed us to shed light into the molecular details of the interaction of the fusion peptide with a membrane bilayer at different pH values. These simulations revealed that pH has a strong effect on the peptide behavior. At low pH (3–5), the N-terminus and the side chains of Glu11 and Asp19 are predominantly protonated, which stabilizes the vertical membrane-spanning configuration tested in this work. In this pH range, the peptide also forms frequent interactions with the lipid phosphate and ester oxygens. On the other hand, at higher pH (7–9) the peptide has a lower tendency to maintain the vertical configuration and adopts a more horizontal water-exposed configuration, interacting less frequently with the lipid ester groups. These findings may explain the twofold activity increase observed at pH 5, since it has previously been proposed that the vertical membrane-spanning configuration is relevant for fusion^7,14–16^.

We note that the effect of pH on the influenza fusion peptide properties and membrane-interaction is a rather complex one. Our results indicate that the protonation of Asp19 changes considerably between pH 5 and 7, which seems to drive the peptide to adopt different configurations in the membrane: membrane-spanning at low pH and more horizontal and shallow configurations at higher pH. This in turn can alter the interaction of the peptide with the membrane. As an example, the radial distribution function and hydrogen bond analysis show that in the simulation set V the N-terminus interacts more with the ester oxygens at low pH than at high pH. This is due to the fact that, besides being more frequently protonated at low pH (as revealed by the titration curves), in acidic conditions, in which the vertical membrane-spanning conformation is stabilized, the N-terminus can also penetrate deeper into the membrane and interact with the ester groups. Thus, although Asp19 does not have frequent interactions with the lipids, as shown in Figs. 9 and 11, the protonation of this residue affects the insertion of the peptide in the membrane, which in turn affects the peptide-lipid interaction profile.

The cpHMD simulations also showed that interactions between the N-terminal region and the lipid oxygens play a very important role in lipid tail protrusion, which has previously been shown to increase in the presence of the influenza fusion peptide and lower the barrier for membrane fusion^19^. This highlights the importance of the N-terminal region for the peptide’s activity, in line with previous works, which proposed that the N-terminus is crucial for fusion^10,15,27^, and that mutations in this residue tend to decrease or abolish the peptide’s fusogenicity^4^. Recently, Worch et al. compared variants of the influenza fusion peptide with free vs acetylated N-terminus, using experimental and computational methods, and observed that a free N-terminus (which tends to be protonated and charged) results in a higher fusogenic activity^15^. They also observed that the 23-residue long fusion peptide with a free N-terminus adopts a more closed helical hairpin conformation, deeply buried within the membrane with a membrane-spanning orientation and promotes lipid disorder. In contrast, the acetylated counterpart has a more open conformation, locates closer to the surface and has a lower impact on the membrane^15^. Another recent simulation study has shown that the protonation of the N-terminus leads to a deeper penetration into the membrane and contributes directly to membrane fusion^27^. The location of the fusion peptide at the N-terminal end of fusion proteins is a common feature found in most class I fusion proteins, which suggests the interaction between the N-terminus and the host membrane may have an important role in promoting fusion in several viruses. Some class II fusion proteins (e.g. Dengue E protein), which have internal fusion peptides, connected to the protein through both ends, contain polar or charged residues in the fusion peptide flanking region, which may play a similar role to the influenza fusion peptide N-terminus by interacting with the lipid headgroups and promoting fusion.

In the cpHMD simulations we used a DMPC lipid bilayer, which was chosen as model lipid bilayer. Thus, we opted for a well parameterized lipid to ensure that the simulations are accurate. One important aspect that should be taken into account is whether these findings can be extrapolated to other membrane systems, including the POPC:POPE membrane that was used experimentally. Both the DMPC membrane and the POPC:POPE membrane are composed of phospholipids which contain a phosphate and two ester groups. Additionally, both membranes are also in the liquid crystalline phase at biologically relevant temperatures of this study and they are both composed by zwitterionic lipids. Given that the relevant interactions observed in our simulations are established between the fusion peptide residues (particularly the N-terminal and Trp14) and the lipid phosphate and ester groups, it is most likely that the same type of interactions occur in the POPC/POPE membrane. In fact, in a recent study^27^, the effect of the influenza fusion peptide on membranes with different compositions was studied using multiscale simulations and no significant differences were observed between pure POPC, POPC:POPE and POPC:DOPE membranes, indicating that the peptide has a similar effect in all these membranes. Interestingly the peptide’s ability to promote fusion of the different membranes was considerably more pronounced when the N-terminal was charged than when it was neutral, which is in agreement with our results. This study clearly indicates that the effect of the peptide on the membrane is not heavily dependent on membrane composition, as long as membranes have similar chemical groups, which can interact with the peptide, particularly with the N-terminal residue. Nevertheless, in the future, it would be interesting to perform cpHMD simulation using different membrane bilayers to test whether there are more subtle differences between different systems.

## Supplementary information

Supplementary information.

Supplementary information.

Supplementary information.

Supplementary information.

## Data Availability

The datasets generated during and/or analyzed during the current study are available from the corresponding author on reasonable request. The simulation software MEAD, meadTools, PETIT and an altered version of GROMACS (with ionic strength as an external parameter) is available in the webpage www.itqb.unl.pt/labs/molecular-simulation/in-house-software. The CpH MD package is made available upon request.
