# Supplementary material for: Effect of pH on the influenza fusion peptide properties unveiled by constant-pH molecular dynamics simulations combined with experiment
Source: Sci Rep. 2020 Nov 18;10:20082. doi: 10.1038/s41598-020-77040-y (PMC7674464; doi:10.1038/s41598-020-77040-y)
Supplement: Supplementary file 1 — Supplementary information. [file 41598_2020_77040_MOESM1_ESM.pdf]

Supporting Information

Effect of pH on the influenza fusion peptide properties unveiled by constant-pH molecular dynamics simulations combined with experiment

Diana Lousa[1]*, Antónia R. T. Pinto[2], Sara R. R. Campos[1], António M. Baptista[1], Ana S. Veiga[2], Miguel A. R. B. Castanho[2] and Cláudio M. Soares[1]*

[1] ITQB NOVA, Instituto de Tecnologia Química e Biológica António Xavier, Universidade Nova de Lisboa, Av. da República, 2780-157 Oeiras, Portugal

[2] Instituto de Medicina Molecular, Faculdade de Medicina de Lisboa, 1649-028 Lisboa, Portugal

Supporting Materials and Methods

Constant-pH MD simulation setup

Before performing the constant-pH MD simulations, the systems were subjected to energy minimization and initialization protocols. First, a rigid PB/MC calculation was carried out to assign initial protonation states to each system, according to the corresponding pH value. An energy minimization step was then performed, using 2000 steps of steepest descent with position restraints on all the peptide heavy atoms, followed by 2000 steps of steepest descent with position restraints on the peptide Cα atoms and another 2000 steps without position restraints. The initialization stage comprised three MD steps using the same setup that was used for the MD blocks performed in the CpH simulations, unless otherwise specified. In the first step, initial velocities were randomly generated from a Maxwell-Boltzmann distribution, using different random seeds for each replicate and the system was simulated for 50 ps in the NVT ensemble, with a temperature coupling constant of 0.01 ps and position restraints in the peptide heavy atoms. In the second step, a 50 ps-long simulation was performed, setting the temperature coupling constant to 0.1 ps and applying position restraints to the peptide Cα atoms. In the third step, the system was simulated for 100 ps in the NPT ensemble, with the same temperature coupling constant, a pressure coupling constant of 0.5 ps and no position restraints.

The output of the last initialization step was used to initiate the constant pH MD simulations, in which 5 sites were considered protonable, namely the N-terminal site of Gly1, the side chain carboxylic group of Glu11, the side chain hydroxyl group of Tyr22, the side chain carboxyl group of Asp19 and the C-terminal carboxyl group of Ser24. A reduced titration approach was used to reduce the computational cost, as described in previous works.[1-4]

MD simulations with fixed residue protonation states

Standard MD simulations with fixed protonation states were performed with GROMACS version 2018.3. These simulations started from the final structure obtained in replicate 5 of the CpH MD simulation set V performed at pH 7. In this CpH simulation, the tilt angle decreased and the peptide moved towards one of the membrane leaflets (movie_S1 in supporting information). However, the simulation time was not sufficient for the peptide to loose contact with the other leaflet and become horizontal. Thus, we decided to perform standard MD simulations with fixed protonation states, using GROMACS 2018 with GPU acceleration, which allowed us to run long trajectories of 12.5 μs. All the titrable residues were deprotonated, since the objective was to simulate the peptide at high pH. The settings used in these simulations were identical to those used in the MD block of CpH MD simulations, with the exception of the cutoff scheme, which in this case was Verlet to allow for GPU acceleration, applying a cutoff of 1.4 nm.

Supporting Figures

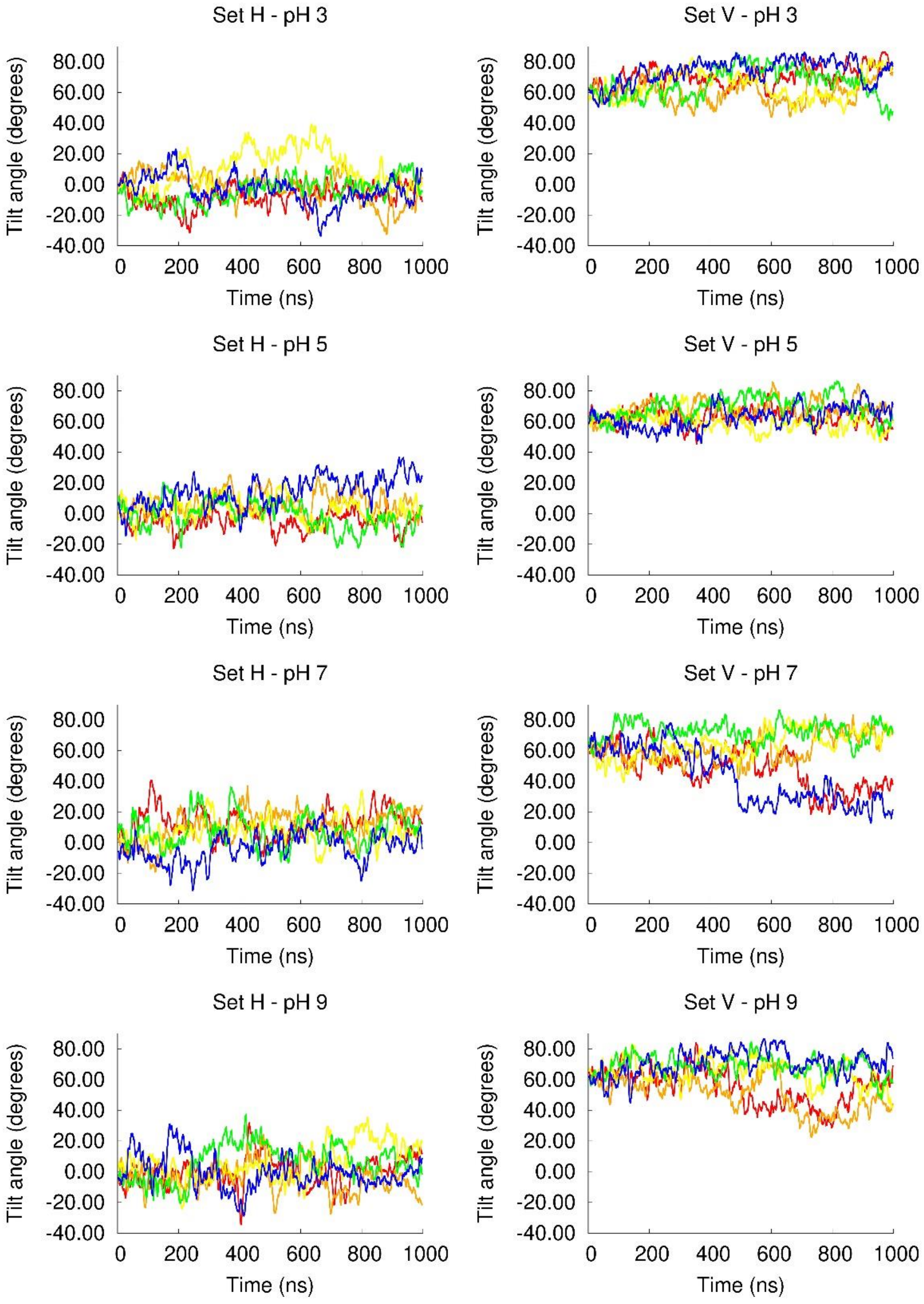

Figure S1 Temporal evolution of the peptide N-terminal helix tilt angle. The tilt angle was computed as the angle of a vector defined by the Cα atoms of residues 1 and 11 and the XY-plane. Each line corresponds to an independent replicate simulation performed in the same conditions

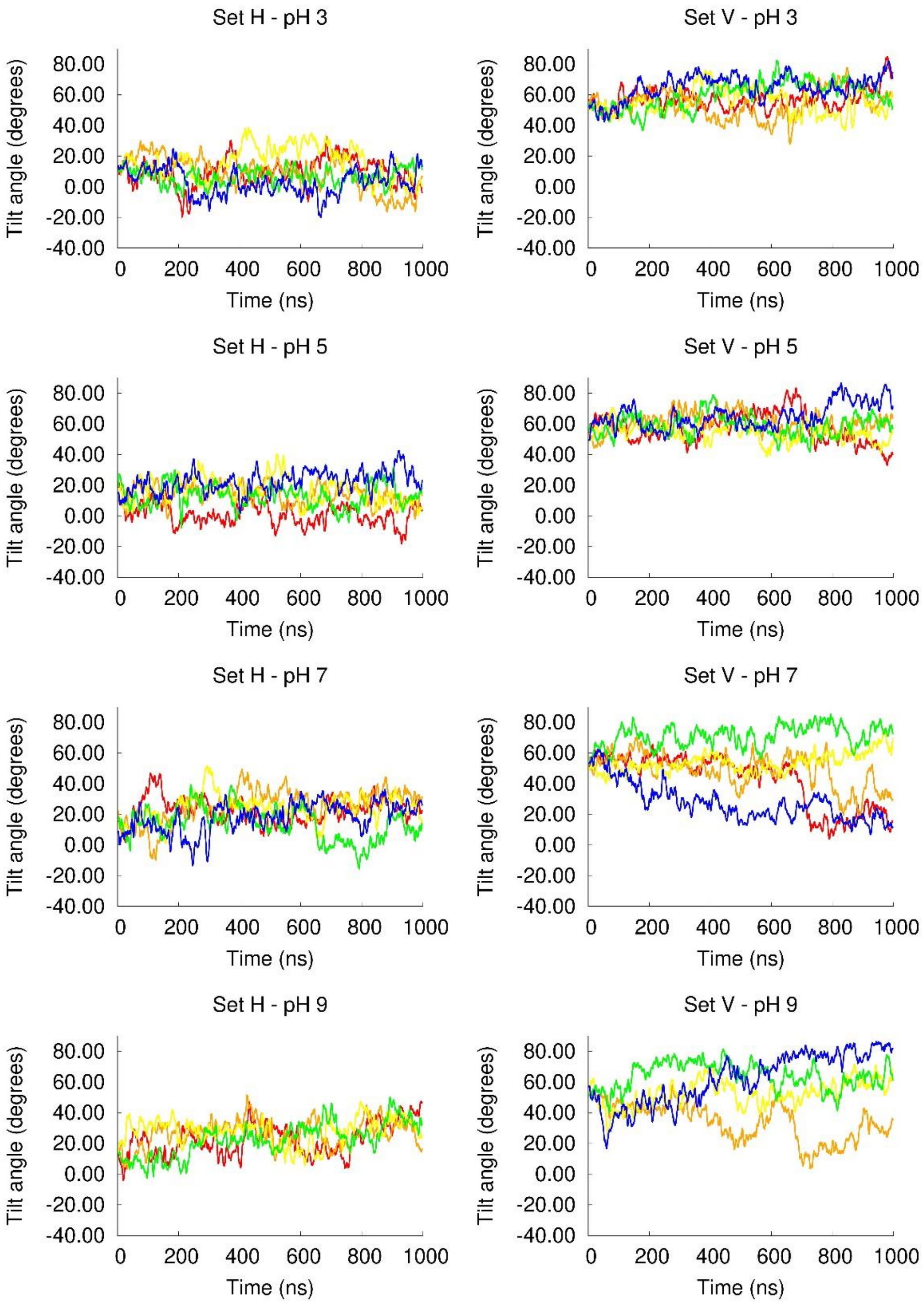

Figure S2. Temporal evolution of the peptide C-terminal helix tilt angle. The tilt angle was computed as the angle between the vector defined by the Cα atoms of residues 15 and 22 and the XY-plane. Each line corresponds to an independent replicate simulation performed in the same conditions. The C-terminal helix tilt angle of the replicates in which this helix unfolded at pH 9 was not calculated (replicates 5 in set H and 1 in set V).

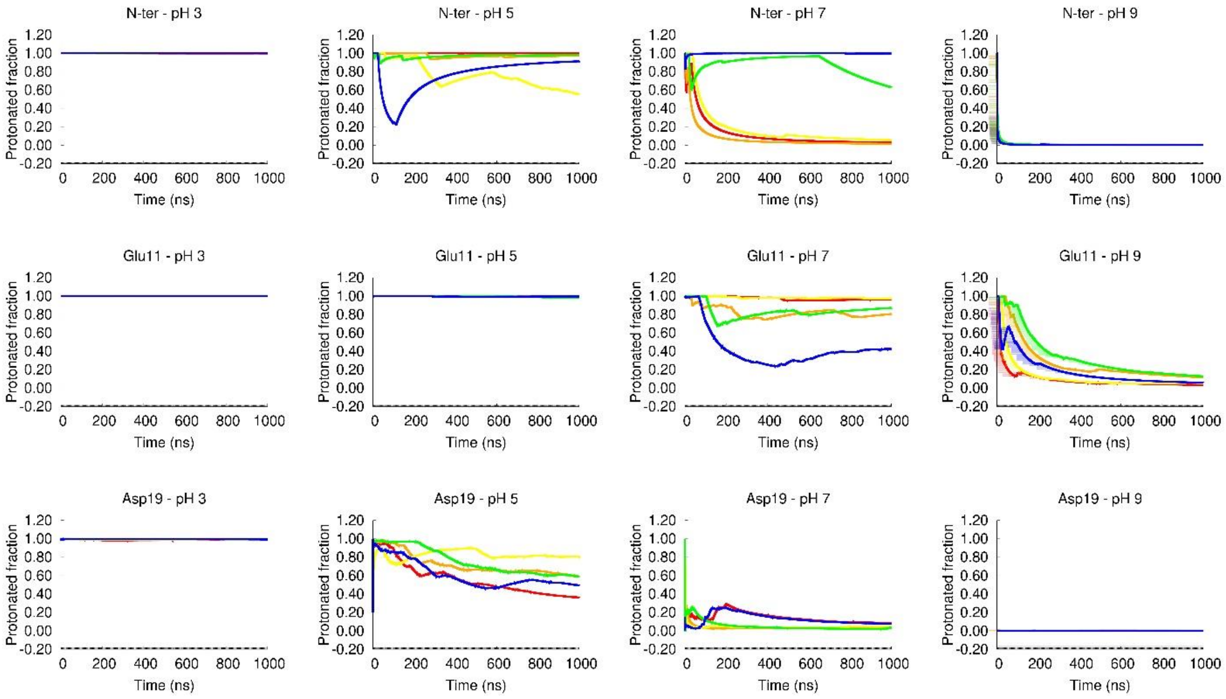

Figure S3. Cumulative protonation averages of the N-terminal (top row), Glu11 (middle row) and Asp19 (bottom row) at each simulated pH value in the simulation set H. Each line corresponds to an independent replicate simulation.

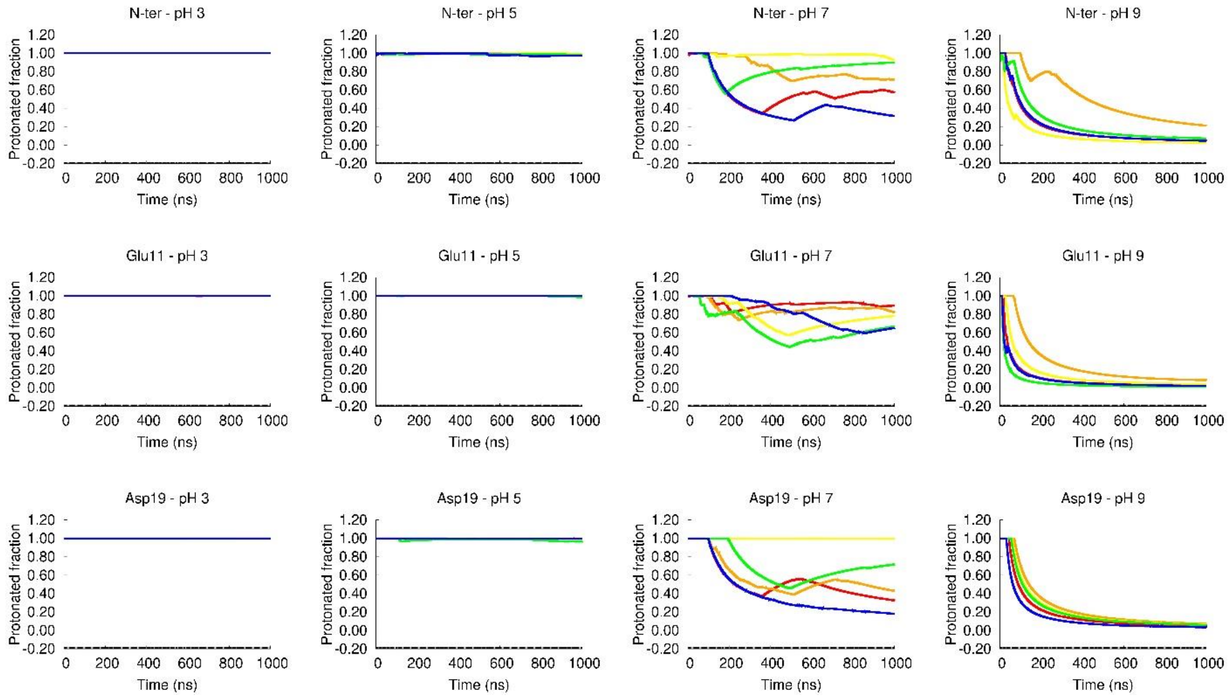

Figure S4. Cumulative protonation averages of the N-terminal (top row), Glu11 (middle row) and Asp19 (bottom) row at each simulated pH value in the simulation set V. Each line corresponds to an independent replicate simulation.

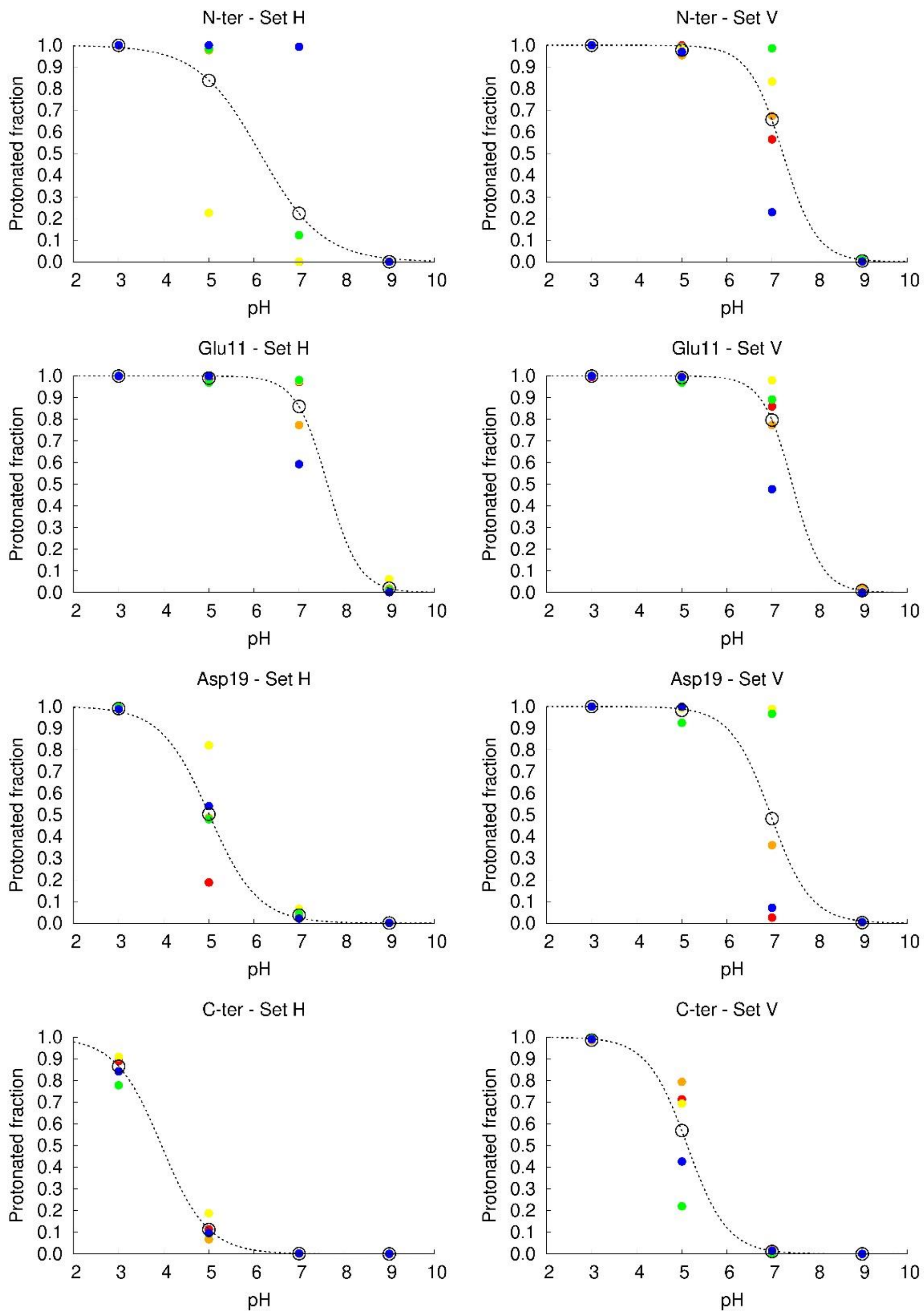

Figure S5. Average protonation values obtained from the last 400 ns of simulation. The filled circles correspond to independent replicates, the empty circles correspond to the average value across all replicates at each pH and the dashed lines represent the corresponding Hill curve fits.

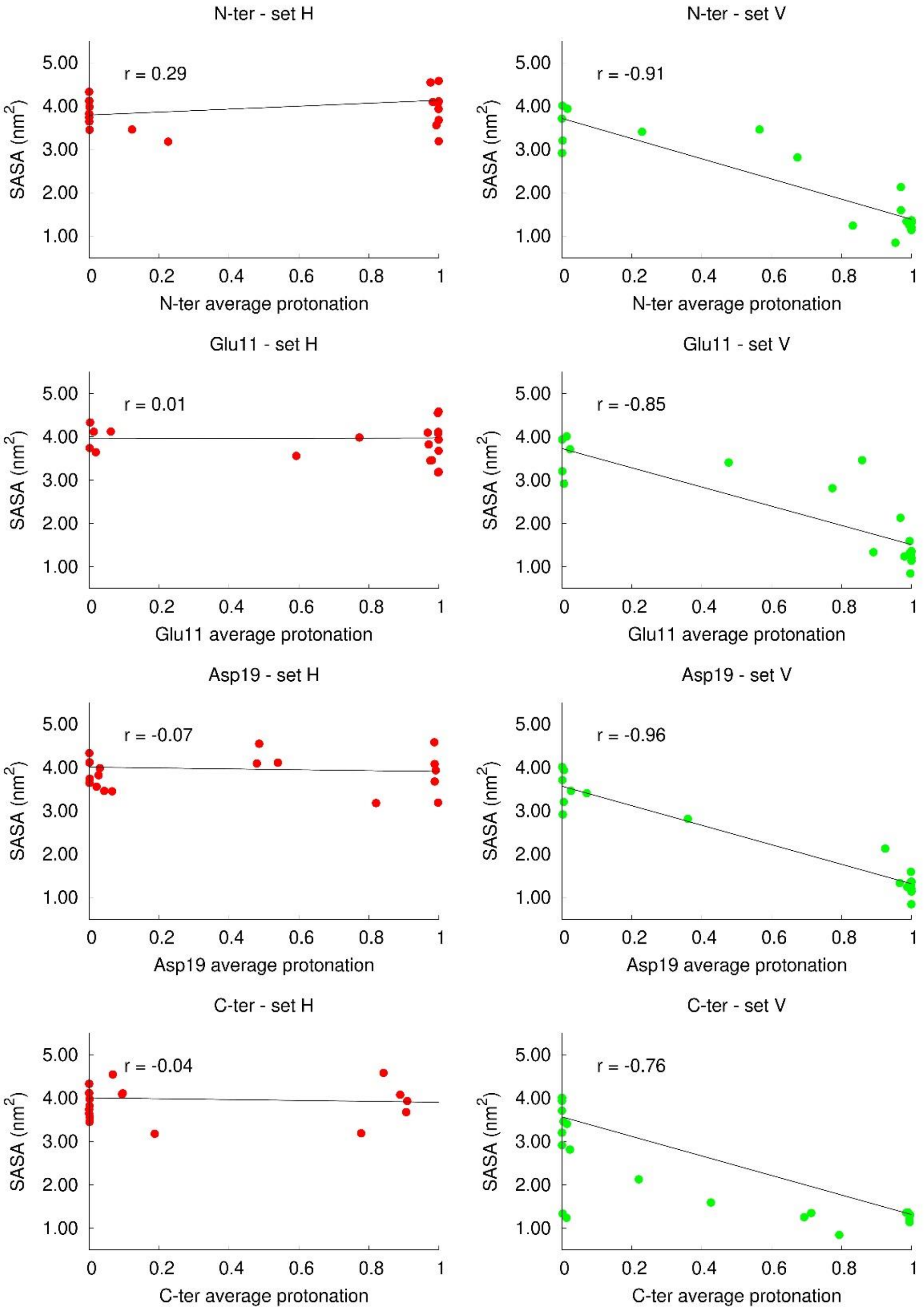

Figure S6. Scatter plots of the FP solvent accessible surface area (SASA) vs average protonation of each residue for the simulation set H (left side) and V (right side). Each point represents one independent replicate simulation at a given pH value, the black line corresponds to the best linear fit to the points and the r value indicates the statistical correlation. The average SASA and protonation was calculated over the last 400 ns of simulation for each replicate.

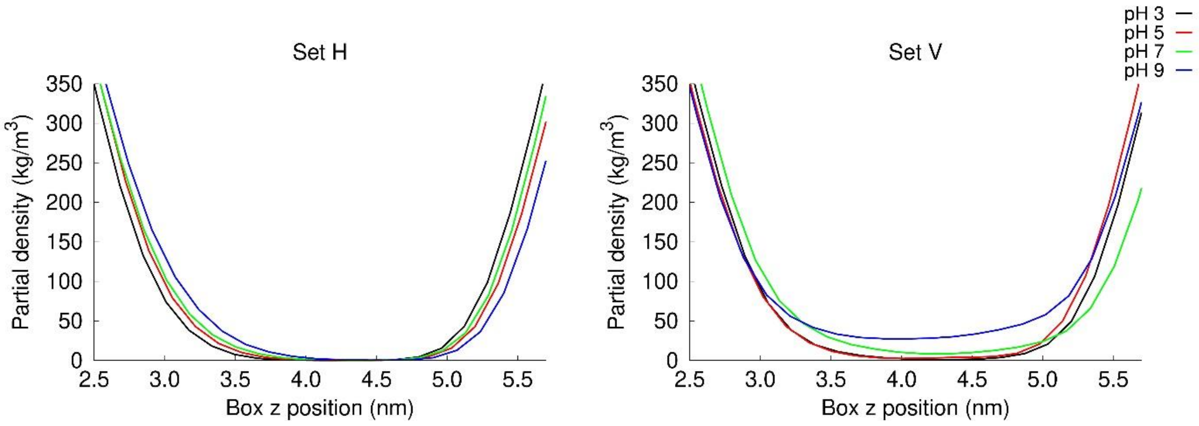

Figure S7. Water density as a function of position along the Z-axis for the simulation set H (left side and V (right side). Each line corresponds to one of the simulated pH values and the partial density was calculated using the GROMACS tool *gmx_density* over the last 400 ns of all the replicate simulations performed at a given pH value.

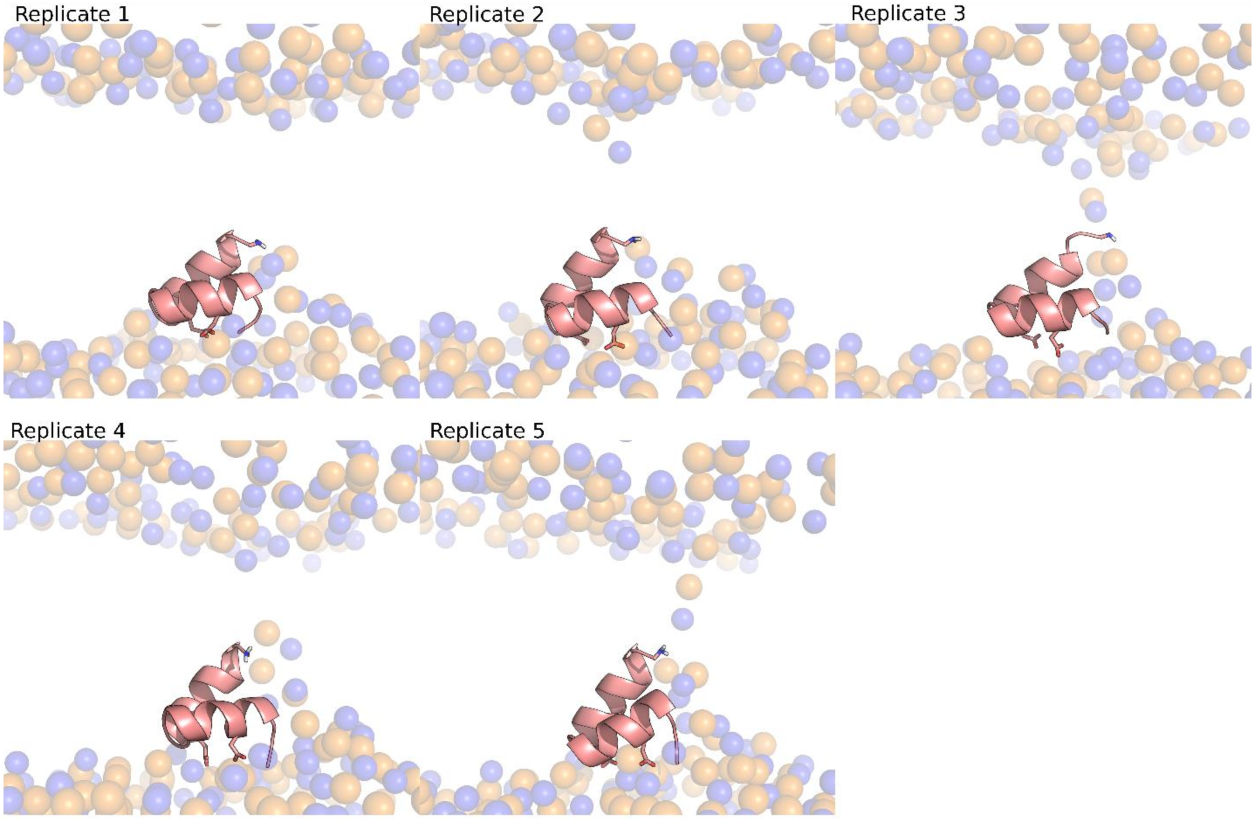

Figure S8. Final conformations of the FP after 12.5 μs of standard MD simulation with fixed protonation states. Each panel shows the final conformation of one of the replicate MD simulations performed with GROMACS version 2018.3 with all the protonable residues deprotonated. The molecular images of the final conformations were built with PyMOL[5] The color code used in the figure is identical to the one described in Figure 3.

a

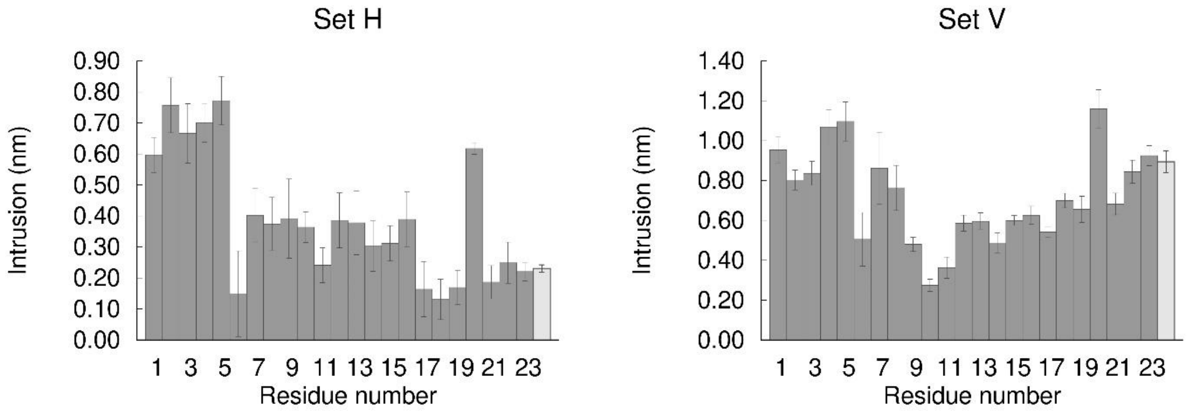

b

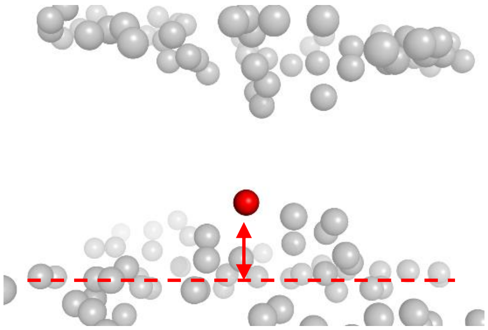

Figure S9. Lipid phosphate intrusion. The plots (a) show the average phosphate displacement in the z direction relative to the average phosphate position in the respective leaflet, for the lipids that are interacting with each residue, as illustrated in scheme (b). The intrusion values correspond to the average obtained across all replicates and the error bars were calculated using a bootstrap method: bootstrap method: for each residue, five new values were resampled with replacement from the original set of five replicates and their mean value was computed, this process was repeated 1000 times and the error was calculated as the standard deviation of this 1000 mean values.. The flanking residue Ser24 is displayed in light grey to highlight the fact that this residue corresponds to a flanking residue and is not part of the fusion peptide itself. The molecular image shown in panel b was built with PyMOL[5].

movie_S1. This movie shows the peptide deviating from the vertical conformation. The trajectory was obtained from replicate 1 at pH 7 in the simulation set V and spans for 1 μs with an interval of 1 ns between snapshots. The FP is shown using a cartoon representation colored in pink, with the N-terminus, Glu11 and Asp19 highlighted in sticks, and the membrane surface is displayed in golden with transparency.

start_H.pdb: Structure used to initiate the simulation set H. This structure was obtained in a previous work[6] where self-assembly MD simulations were used to study the influenza fusion peptide interaction with a lipid membrane and corresponds to the final conformation of replicate 4 simulated in that work.

start_V.pdb: Structure used to initiate the simulation set V. This structure was obtained in a previous work where self-assembly MD simulations were used to study the influenza fusion peptide interaction with a lipid membrane and corresponds to the final conformation of replicate 1 simulated in that work.

References

1. Campos, S.R.R., Iranzo, O. & Baptista, A.M. Constant-pH MD Simulations Portray the Protonation and Structural Behavior of Four Decapeptides Designed to Coordinate Cu2+. *J. Phys. Chem. B* **120**, 1080-1091 (2016).

2. Carvalheda, C.A., Campos, S.R.R., Machuqueiro, M. & Baptista, A.M. Structural effects of pH and deacylation on surfactant protein C in an organic solvent mixture: A Constant-pH MD study *J. Chem. Inf. Model.* **53**, 2979-2989 (2013).

3. Machuqueiro, M. & Baptista, A.M. Constant-pH molecular dynamics with ionic strength effects: Protonation-conformation coupling in decalysine. *J. Phys. Chem. B* **110**, 2927-2933 (2006).

4. Machuqueiro, M. & Baptista, A.M. Acidic range titration of HEWL using a constant-pH molecular dynamics method. *Proteins: Struct., Funct., Bioinf.* **72**, 289-298 (2008).

5. DeLano, W.L. The PyMOL molecular graphics system; DeLano Scientific LLC: Palo Alto, CA, USA. http://www.pymol.org (2002).

6. Victor, B.L., Lousa, D., Antunes, J.M. & Soares, C.M. Self-assembly molecular dynamics simulations shed light into the interaction of the influenza fusion peptide with a membrane bilayer. *J. Chem. Inf. Model.* **55**, 795-805 (2015).
